# *Glucan Synthase-like 2* is Required for Seed Initiation and Filling as Well as Pollen Fertility in Rice

**DOI:** 10.1186/s12284-023-00662-z

**Published:** 2023-10-07

**Authors:** Ronghua Qiu, Yang Liu, Zhengzheng Cai, Jieqiong Li, Chunyan Wu, Gang Wang, Chenchen Lin, Yulin Peng, Zhanlin Deng, Weiqi Tang, Weiren Wu, Yuanlin Duan

**Affiliations:** 1 Key Laboratory of Ministry of Education for Genetics, Breeding and Multiple Utilization of Crops, Fujian Agriculture and Forestry University, Fuzhou, 350002 China; 2 Fujian Key Laboratory of Crop Breeding By Design, Fujian Agriculture and Forestry University, Fuzhou, 350002 China

**Keywords:** Rice, Callose, *Glucan Synthase*, Pollen fertility, Seed initiation

## Abstract

**Background:**

The *Glucan synthase-like* (*GSL*) genes are indispensable for some important highly-specialized developmental and cellular processes involving callose synthesis and deposition in plants. At present, the best-characterized reproductive functions of *GSL* genes are those for pollen formation and ovary expansion, but their role in seed initiation remains unknown.

**Results:**

We identified a rice seed mutant, *watery seed 1-1* (*ws1-1*), which contained a mutation in the *OsGSL2* gene. The mutant produced seeds lacking embryo and endosperm but filled with transparent and sucrose-rich liquid. In a *ws1-1* spikelet, the ovule development was normal, but the microsporogenesis and male gametophyte development were compromised, resulting in the reduction of fertile pollen. After fertilization, while the seed coat normally developed, the embryo failed to differentiate normally. In addition, the divided endosperm-free nuclei did not migrate to the periphery of the embryo sac but aggregated so that their proliferation and cellularization were arrested. Moreover, the degeneration of nucellus cells was delayed in *ws1-1*. *OsGSL2* is highly expressed in reproductive organs and developing seeds. Disrupting *OsGSL2* reduced callose deposition on the outer walls of the microspores and impaired the formation of the annular callose sheath in developing caryopsis, leading to pollen defect and seed abortion.

**Conclusions:**

Our findings revealed that *OsGSL2* is essential for rice fertility and is required for embryo differentiation and endosperm-free nucleus positioning, indicating a distinct role of *OsGSL2*, a callose synthase gene, in seed initiation, which provides new insight into the regulation of seed development in cereals.

**Supplementary Information:**

The online version contains supplementary material available at 10.1186/s12284-023-00662-z.

## Background

Endosperm is the main component of cereal grain and the primary source of calory for humans. Endosperm development is initiated by the fertilization of two central cells with one sperm in the embryo sac, followed by a process of five stages: coenocyte (or free nuclei) generation, cellularization (cell wall formation), cell differentiation, grain filling, and maturation (Olsen 2004; Sabelli and Larkins 2009). In rice, these five stages take place in the first 2 days after pollination (DAP), from 3 to 5 DAP, from 6 to 9 DAP, from 6 DAP onwards, and by 21 DAP, respectively (Wu et al. 2016b). The coenocyte and cellularization stages are comparatively shorter, but determine the cell number and lay the foundation for grain filling (Dante et al. 2014). The rate and duration of endosperm proliferation during the syncytial phase are important determinants of final seed size (Garcia et al. 2005), and the timing of endosperm cellularization affects grain yield (Olsen 2004). The initial phase plays a key role in cereal endosperm development, but its genetic regulation program remains largely unknown (Olsen 2020). Embryo and endosperm can interact during different developmental stages in plants (Nowack et al. 2006). Endosperm is essential for embryogenesis with the function of nourishing the early embryo (Olsen and Becraft 2013; Zheng and Wang 2015).

Callose is a β-1,3-linked homopolymer of glucose residues with some β-1,6-branches. Callose is accumulated mainly in the special cell wall and cell wall-associated structures, is involved in a wide range of biological processes, and executes important functions in development and stress responses in plants (Hong et al. 2001; Chen and Kim 2009; Thiele et al. 2009; Zavaliev et al. 2011; Nedukha 2015; Wang et al. 2022). During reproductive development, callose is synthesized and deposited to the cell plate, cell wall, and other locations within microsporocytes and megaspores, functioning as a molecular or nutritional filter and a physical barrier to temporarily isolate the microsporocytes and megaspores from the influence of surroundings (Wang et al. 2004; Chen and Kim 2009; Shi et al. 2016). Callose between the primary cell wall and the plasma membrane can maintain the morphology of the microspore mother cell and prevent the fusion and aggregation of tetrad cells during microspore development (Dong et al. 2005; Chen et al. 2009; Wang et al. 2020). Accurate deposition and degradation of callose are crucial for normal reproductive development. Either excessive or insufficient callose and premature or delayed degradation of callose will lead to sterility in flowering plants (Franklin-Tong 1999; Dong et al. 2005; Albert et al. 2011; Qin et al. 2012; Liu et al. 2015; Song et al. 2016; Zhang et al. 2018; Somashekar et al. 2023). Therefore, callose is essential for sporogenesis and male/female gametophyte formation in plants.

Callose is synthesized by plasma membrane-located callose synthase (CalS; also referred to as glucan synthase-like, GSL) complex (Verma and Hong 2001). *GSL* genes widely exist in various plant species, and each species contains multiple members. There are 12 and 10 *GSL* genes predicted in *Arabidopsis* and rice, respectively (Verma and Hong 2001; Yamaguchi et al. 2006). Studies on the function of *GSL* genes in *Arabidopsis* have been focused on pollen formation. Five out of the 12 *AtGSL* genes (*CalS5/GSL2*, *CalS9/GSL10*, *CalS10/GSL8*, *CalS11/GSL1*, and *CalS12/GSL5*) were shown to be involved in microsporogenesis and male gametophyte development (Shi et al. 2016; Wang et al. 2020). *AtGSL1* and *AtGSL5* are related to the formation of callose walls in the tetrad, and AtGSL1 might act as an auxiliary protein to assist AtGSL5 (Enns et al. 2005). *AtGSL2* is specifically responsible for callose deposition in the pollen mother cell and pollen tube (Dong et al. 2005; Nishikawa et al. 2005). *AtGSL8* and *AtGSL10* are independently required for asymmetric microspore division and the entry of microspores into mitosis (Töller et al. 2008). In rice, *OsGSL5*, a homolog of *AtGSL2*, was also found to be responsible for pollen development (Shi et al. 2015). The protein OsGSL5 is located on the plasma membrane of pollen mother cells and is responsible for the biogenesis of callose in anther locules through premeiotic and meiotic stages (Somashekar et al. 2023).

The above studies have revealed that *GSL* genes play critical roles in pollen formation and fertility. Besides, *GSL* genes have also been found to play roles in other developmental processes, including stomatal patterning (Guseman et al. 2010), hypocotyl tropic response (Han et al. 2014), inflorescence growth (Barratt et al. 2011), leaf vein development (Slewinski et al. 2012), and so on. The information indicating the role of *GSL* genes in the female reproductive process and seed formation is still very limited (Chen et al. 2009; Janas et al. 2022). *CRR1*/*OsGSL8* is the only well-characterized *GSL* gene that plays a pivotal role in seed development, which determines the initial expansion of the ovary by affecting vascular cell patterning in rice (Song et al. 2016).

In this study, we identified a rice mutant *ws1-1*, which showed reduced fertile pollens and produced seeds without embryo and endosperm but filled with transparent liquid. We found that the *OsGSL2* gene in *ws1-1* was disrupted, which reduced the callose deposition on the outer walls of the microspore and disrupted the annular callose sheath in developing caryopsis, so as to impact microsporogenesis and seed development except for seed coat formation. These findings revealed that *OsGSL2* is essential for fertility and seed initiation in rice, suggesting a distinct role in embryo differentiation and seed filling, thus providing insights for regulating seed production in crops.

## Results

### *ws1-1* Produced Seeds Filled with Sucrose Liquid Without Starch

**Fig. 1:**
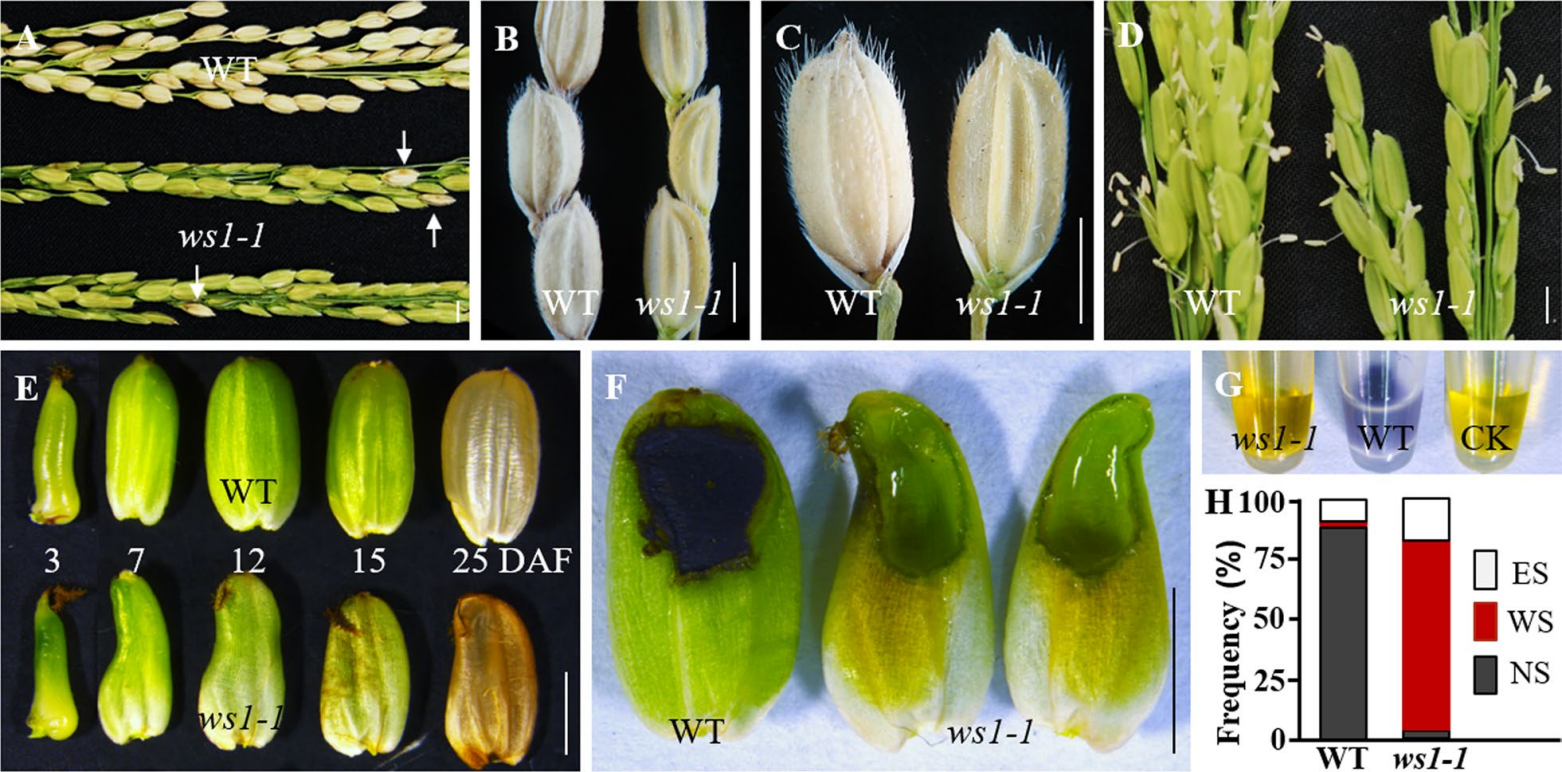
The phenotype of *ws1-1* mutant. **A**–**C** Comparison of wild-type (WT) and *ws1-1* panicles (**A**) and spikelets (**B**, **C**) at the mature stage. The white arrows (in **A**) indicate the starchy seeds in *ws1-1*. **D** Comparison of WT and *ws1-1* panicles at the heading stage. **E** Morphology of seeds produced by WT and *ws1-1* at different DAF. **F**, **G** Starchy endosperm in WT seeds and transparent liquid in *ws1-1* seeds at 12 DAF stained with I_2_-IK solution. **H** Cumulative percentages of normal seeds (NS), watery seeds (WS), and empty seeds (ES, containing no developed caryopsis) in WT and *ws1-1* at the mature stage were obtained from statistics of 30 panicles each. Scale bars = 2 mm

During tillering and flowering stages, *ws1-1* plants exhibited no obvious differences from Nipponbare (wild-type, WT) plants (Additional file 1: Fig. S1). The number and appearance of spikelets in *ws1-1* were normal, but its seed setting rate was extremely low (< 2%; in comparison, the seed setting rate in WT was 90.8%; Fig. 1A–C). At anthesis, anthers dehisced to release mature pollen grains normally in *ws1-1* (Fig. 1D). After anthesis, seed coats developed normally in length in *ws1-1*, but the upper part of caryopsis became apparently narrower from ~ 3 DAP until maturity (Fig. 1E). Besides, unlike WT seeds, most (~ 75%) *ws1-1* seeds did not contain milky or starchy endosperm but were filled with transparent liquid (Fig. 1F, G), which was rich in sucrose and other kinds of soluble sugar (Additional file 1: Fig. S2) and was eventually dried, leaving shrunken and inviable seeds at maturity. These results indicated that starch could not be synthesized and accumulated in *ws1-1* seeds, but this defect was not due to the shortage of sugar supply. Occasionally, watery seeds were also observed in WT, but the rate was very low (< 1%; Fig. 1H).

Observation of the transverse sections of WT and *ws1-1* caryopses at different developmental stages showed that the difference between *ws1-1* and WT seeds was not visible at 1 DAP (Additional file 1: Fig. S3A, B), but gradually became obvious from 3 DAP onwards (Additional file 1: Fig. S3E–L). At later developmental stages, a WT caryopsis was filled with endosperm, while a mutant caryopsis enlarged without forming endosperm but remained hollow inside (Additional file 1: Fig. S3K, L). These results indicated that endosperm failed to develop from the early stage of seed development in *ws1-1*.

### Anther Development and Microsporogenesis were Defective in *ws1-1*

To find out the causes of low seed setting rate in *ws1-1*, we examined the functions of *ws1-1* male and female gametophytes by performing reciprocal crosses between WT and *ws1-1*, with artificial and natural selfings as controls. The seed setting rate of artificial selfing was similar to that of natural selfing in both WT and *ws1-1*, indicating that the operation of artificial pollination had little influence on the seed setting rate in our experiment. Both WT × *ws1-1* and *ws1-1* × WT yielded an intermediate seed setting rate, which was much higher than that of *ws1-1* selfing (< 2%) but much lower than that of WT selfing (~ 90%; Additional file 1: Fig. S4). The results of reciprocal crosses indicated that the functions of male gametophyte and female gametophyte are both compromised in *ws1-1*, suggesting that *WS1* is probably required for the development of both male and female gametophytes.

**Fig. 2:**
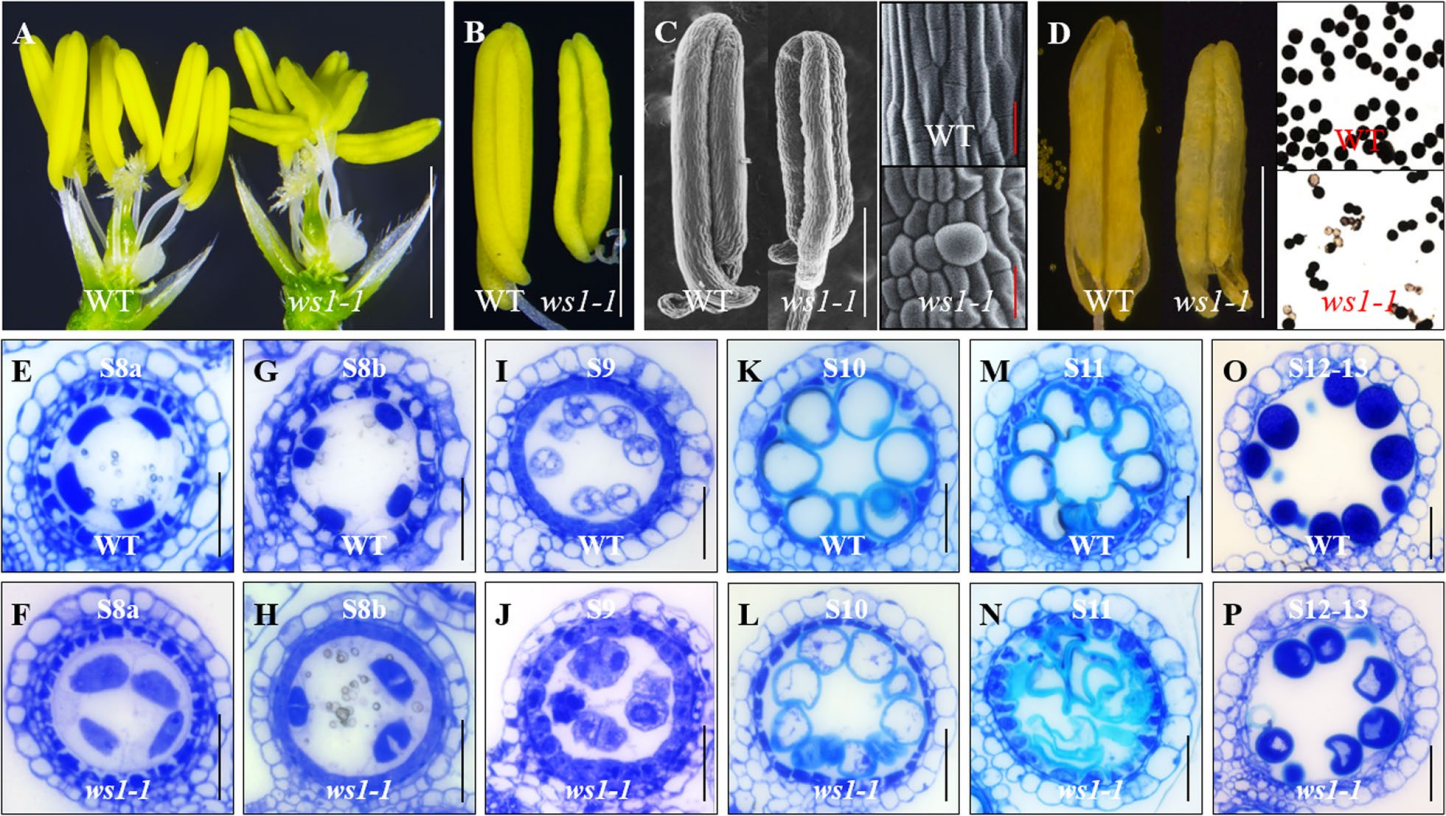
Male reproductive development was defective in *ws1-1.* **A** Spikelets of WT and *ws1-1* with lemma/palea removed. **B** Anthers of WT and *ws1-1*. **C** SEM imagines of WT and *ws1-1* anthers. **D** Comparison of WT and *ws1-1* anthers and pollens at the mature stage; the pollens were stained with I_2_-IK solution (normal pollens showed dark color). **E**–**P** Comparison of microsporogenesis in WT and mutant anthers by staining of transverse sections with 0.1% toluidine blue. The development stages were described by Zhang et al. (2011). Scale bar = 2 mm in (**A**–**D**, white bars), 100 µm in (**D**, red bars), and 25 µm in (**E**–**P**)

So, we further examined the reproductive development in *ws1-1*. Compared with WT, *ws1-1* had smaller anthers but normal pistils in appearance (Fig. 2A). Occasionally, bilocular anthers were observed in *ws1-1*, while WT anthers always had four chambers (Fig. 2B). SEM images showed that WT anthers had a smooth surface, of which the cells were basically uniform in size and orderly arranged, while the surface of *ws1-1* anthers was uneven and its cells varied significantly in size (Fig. 2C). Anther dehiscence in *ws1-1* was largely normal, but the number of pollen grains per anther was only ~ 35% of that in WT, and only about half of the pollens appeared normal in shape and I_2-_KI staining (Fig. 2D). Collectively, these observations indicated that the *WS1* mutation resulted in anther and pollen defects.

To investigate in detail the cellular defects in *ws1-1* pollen development, we examined its transverse anther sections at different developmental stages according to the previous description (Zhang et al. 2011). Anther development in *ws1-1* was overall normal prior to stage 8 or pollen mother cell meiosis (Additional file 1: Fig. S5). At stage 8, the dyad and tetrad microspores generally displayed a uniform appearance in WT (Fig. 2E, G), but showed irregular morphology and sizes in *ws1-1* (Fig. 2F, H). At stage 9, free spherical microspores were released from the tetrads in WT (Fig. 2I), but in *ws1-1* most microspores adhered together in a disordered manner without clear borders among them (Fig. 2J). WT anthers generated vacuolated round microspores at stage 10 and falcate-shaped microspores at stage 11, whereas *ws1-1* microspores were markedly different from WT microspores in size and shape at these two stages (Fig. 2K–N). Finally, plump pollens were produced in the WT, while most *ws1-1* pollens were deformed or abnormal in size, with obvious reduction or even complete loss of starch granules (Fig. 2O, P). These observations indicated that the microspore abnormality in *ws1-1* began from the dyad stage. Since glucan synthase might also play a role in starch biosynthesis, the reduction in pollen count could potentially be due to reduced starch filling.

### The Initiations of Embryo and Endosperm were Abnormal in *ws1-1*

**Fig. 3:**
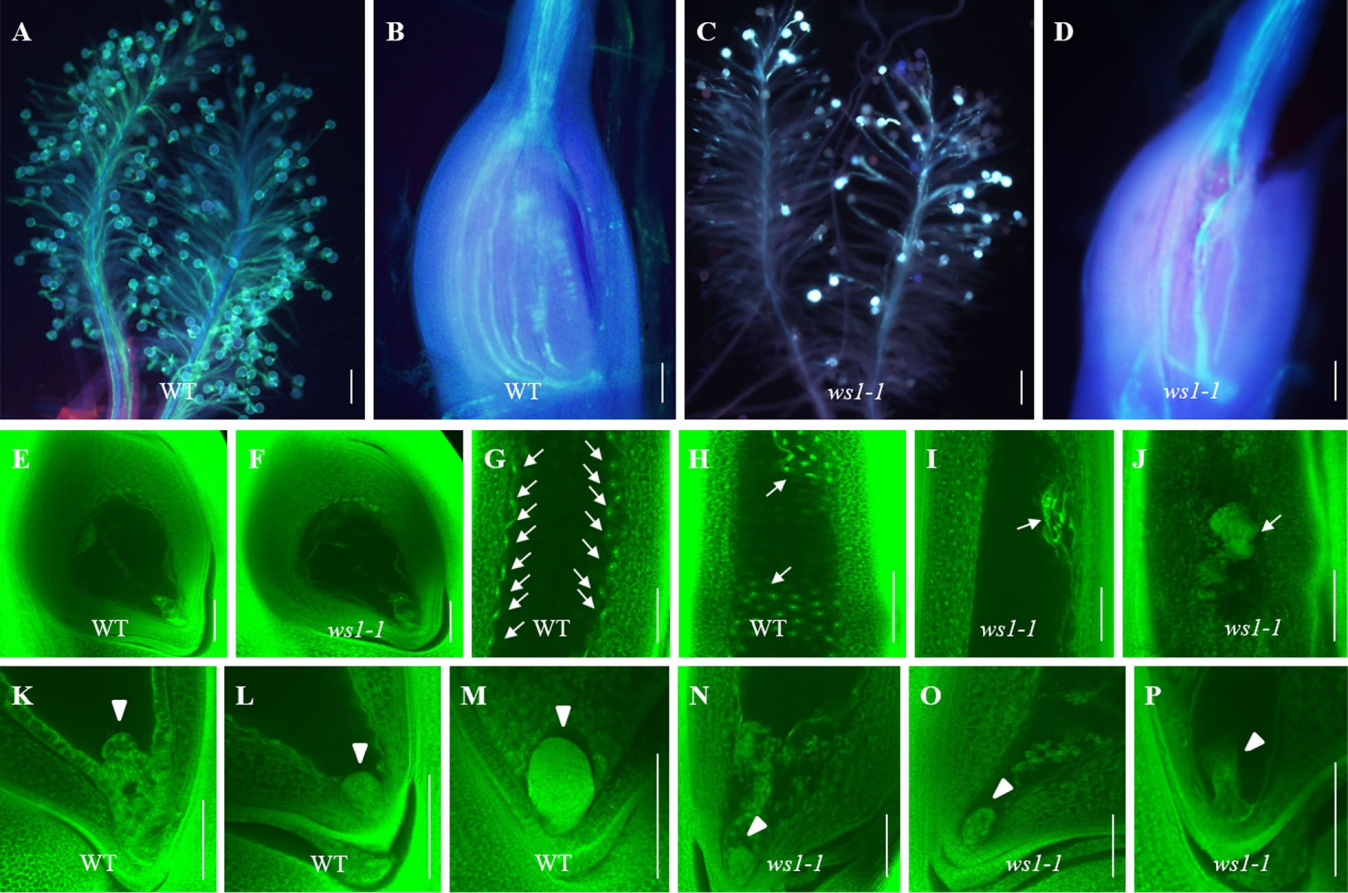
Comparison of the pollination, fertilization and early seed development between the WT and *ws1-1*. **A**–**D** Pollen germination and pollen tube elongation in the WT (**A**, **B**) and *ws1-1* (**C**, **D**). **E**, **F** Mature embryo sac of the WT (**E**) and *ws1-1* (**F**). G, H WT endosperms at 2 DAP (**G**) and 3–5 DAP (**H**). I, J *ws1-1* endosperms at 2 DAP (**I**) and 3–5 DAP (**J**). **K**–**P** The early-stage embryo in WT (**K**–**M**) and *ws1-1* (**N**–**P**). White arrows in **G**–**J** and arrowheads in **K**–**P** indicate endosperms and embryos, respectively. The number of florets that were observed is over 10 for each figure. Scale bar = 100 µm in **A**–**D** or 50 µm in **E**–**P**

To determine whether the abnormal pollen development was the major cause of the low seed setting rate in *ws1-1*, we examined the vigor of *ws1-1* pollens in vivo by manual self-pollination and toluidine blue staining. In the WT, pollen grain germination, and pollen tube elongation until arriving at the nucellus for double fertilization were observed expectedly (Fig. 3A, B). In *ws1-1*, similar phenomena without obvious abnormality were also observed in pollens with normal development, where these normal pollens could adhere to and germinate on the stigma, and pollen tubes could elongate and finally enter nucelli (Fig. 3C, D). These results suggested that the normal *ws1-1* pollens had a similar vitality to WT in vivo, although their number was much smaller. We also examined *ws1-1* ovules by whole-mount staining. There was no significant difference in the structure of the embryo sac between the WT and *ws1-1*, suggesting that the embryo sac developed normally in *ws1-1* (Fig. 3E, F). The above results together indicated that *ws1-1* was normal in fertilization. Therefore, we suspected that its low seed setting rate was probably caused by defects in embryo and/or endosperm development after fertilization.

To verify this hypothesis, we observed WT and *ws1-1* seeds at early developmental stages by whole-mount staining. In a WT embryo sac, as reported (Wu et al. 2016b), endosperm nuclei were divided, rapidly separated, migrated, and distributed to the peripheries of the endosperm cells during 1–2 DAP (Fig. 3G), and then the syncytial endosperm was cellularized during 3–5 DAP (Fig. 3H). In *ws1-1*, the primary endosperm nuclei also proliferated, but they did not separate and failed to migrate to the periphery (Fig. 3I), leading to a clump of endosperm nuclei (Fig. 3J), which stopped the production of endosperm nuclei and was gradually degenerated. Besides, as reported (Wu et al. 2016a), most nucellar cells degenerated during 1–5 DAP in WT (Fig. 3K, L), but there were still multicellular tissue residues in *ws1-1* nucellus (Fig. 3N, O), indicating that the degeneration of nucellus cells was delayed in *ws1-1.*

For embryogenesis, coupling with the process of starch deposition, a WT zygote developed by cell division into a proembryo at 1 DAP (Fig. 3K), and then into a globular embryo at 2 or 3 DAPs (Fig. 3L), and further into a pear-shaped embryo at 4 DAPs (Fig. 3M). In *ws1-1*, a multicellular proembryo was also produced at 1 DAP (Fig. 3N), but the proembryo developed into an abnormal embryo with a smaller size and irregular shape (Fig. 3O), which gradually degenerated and disappeared in the early stage (Fig. 3P).

### *WS1* Encodes a Putative Callose Synthase

**Fig. 4:**
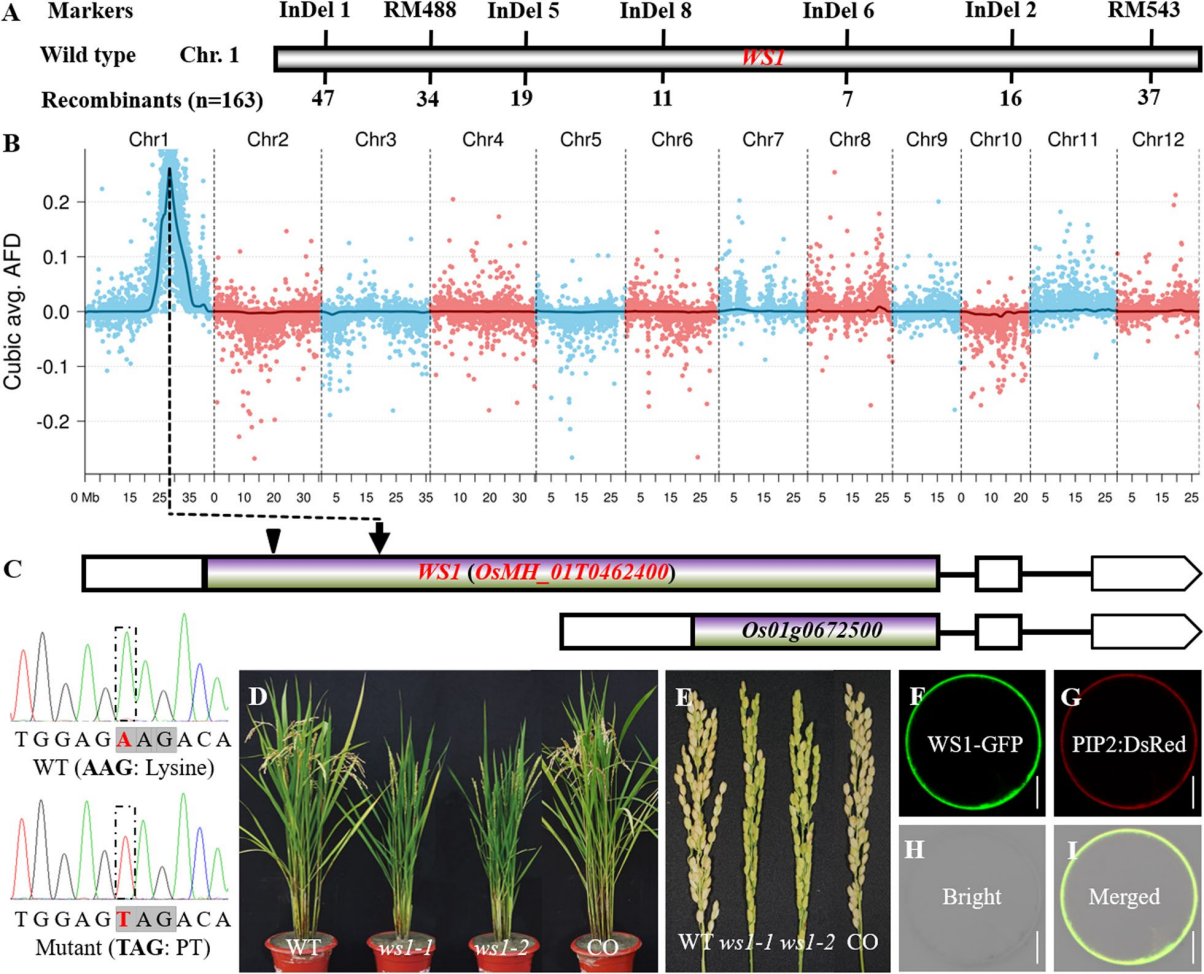
Positional cloning and functional confirmation of *WS1*. **A** Result of *WS1* mapping by linkage analysis. The number of recombinants at each marker is shown below the horizontal bar. *WS1* was mapped to a region between markers InDel6 and InDel8. **B** Results of *WS1* mapping by BSA-seq. The peak tip suggested the most probable position of WS1. **C** Results of candidate gene identification. An SNP (indicated by arrow) was found in *WS1*, with transversion of A to T in *ws1-1*, leading to a change of Lysine codon to a premature stop codon. **D**, **E** Functional validation of *WS1* by genetic complementation and gene editing. CO, genetic complementation plant; *ws1-2*, *WS1* knockout mutant generated by CRISPR/Cas9 editing. The WT phenotype was recovered by genetic complementation in the CO plant, whereas *ws1-2* showed a mutant phenotype similar to *ws1-1*. **F**–**I** *GSL5*CT-GFP was found to be localized to the plasma membrane. WS1-GFP and PIP2:DsRed (Plasma membrane marker) were cotransformed into rice protoplasts. Scale bars = 5 μm

Genetic analysis showed that the WT and mutant phenotypes segregated at a 3:1 ratio in the selfed progeny lines of heterozygotes, suggesting that the mutant phenotype was caused by a single recessive mutation. Linkage analysis based on 163 mutant plants from the F_2_ generation of *ws1-1* × Lemont mapped the *WS1* locus to a 2.18 Mb region between markers InDel6 (29,122,791–29,122,812) and InDel8 (26,939,245–26,939,262) on the long arm of rice chromosome 1 (Fig. 4A). BSA-seq based on F_2:3_ lines verified the result of linkage analysis and indicated that *WS1* was most likely located around the position of 2.18 Mb (Fig. 4B). Sequence comparison showed that there was only an SNP between Nipponbare (A) and *ws1-1* (T) in this region upstream of a gene (*Os01g0672500*) but rather inside a gene according to the annotation in RGAP (http://rice.uga.edu/index.shtml). However, by surveying the annotation in RIGW (http://rice.hzau.edu.cn /rice_rs3/), we found that there was actually a longer ORF (*OsMH_01T0462400*) covering *Os01g0672500* in this region and the SNP (A/T), which resulted in a change from Lysine to a premature stop codon in *ws1-1* (Fig. 4C). So, we thought that *OsMH_01T0462400* was likely to be *WS1*.

To validate the candidate gene, we introduced a complementary plasmid carrying *OsMH_01T0462400* and its promoter into *ws1-1*. The mutant phenotype was rescued and restored to that of WT in all 11 independent transgenic lines (Fig. 4D, E; Additional file 1: Fig. S6). In addition, we also generated 16 independent and effective knockout mutants of *OsMH_01T0462400* from Nipponbare by CRISPR/Cas9, which could be categorized into four alleles, including one-base insertion (470^th^), five-base deletion (461^st^-466^th^), four-base deletion (465^th^-468^th^), and one-base deletion (464^th^). All these alleles were predicted to encode truncated proteins due to frameshifts that led to premature stop codons (Additional file 2: Table S1). All these mutants exhibited the same phenotype as that of *ws1-1* (Fig. 4D, E; Additional file 1: Fig. S6). We named them *ws1-2* to *ws1-5*. The above results confirmed that *OsMH_01T0462400* is *WS1*.

*OsMH_01T0462400/WS1* is predicted to encode a callose synthase named OsGSL2, which contains a conserved β-glucan synthase domain near the 3′-terminal (Additional file 1: Fig. S7) highly similar to other family members in rice and *Arabidopsis* (Yamaguchi et al. 2006). Phylogenetic analysis indicated that *WS1* is closely related to *AtGSL1* and *AtGSL5* (Additional file 1: Fig. S8; Shi et al. 2015). The five mutant alleles, *ws1-1* to *ws1-5*, all encoded non-functional truncated proteins that lacked the crucial callose synthase domain. Therefore, they all were null alleles of *WS1*. Subcellular localization analysis of the GFP-WS1 fusion protein indicated that WS1 is localized in the cell membrane (Fig. 4F–I). This is consistent with the predicted function of *WS1*.

### *WS1* has Higher Expression in Reproductive Organs

**Fig. 5:**
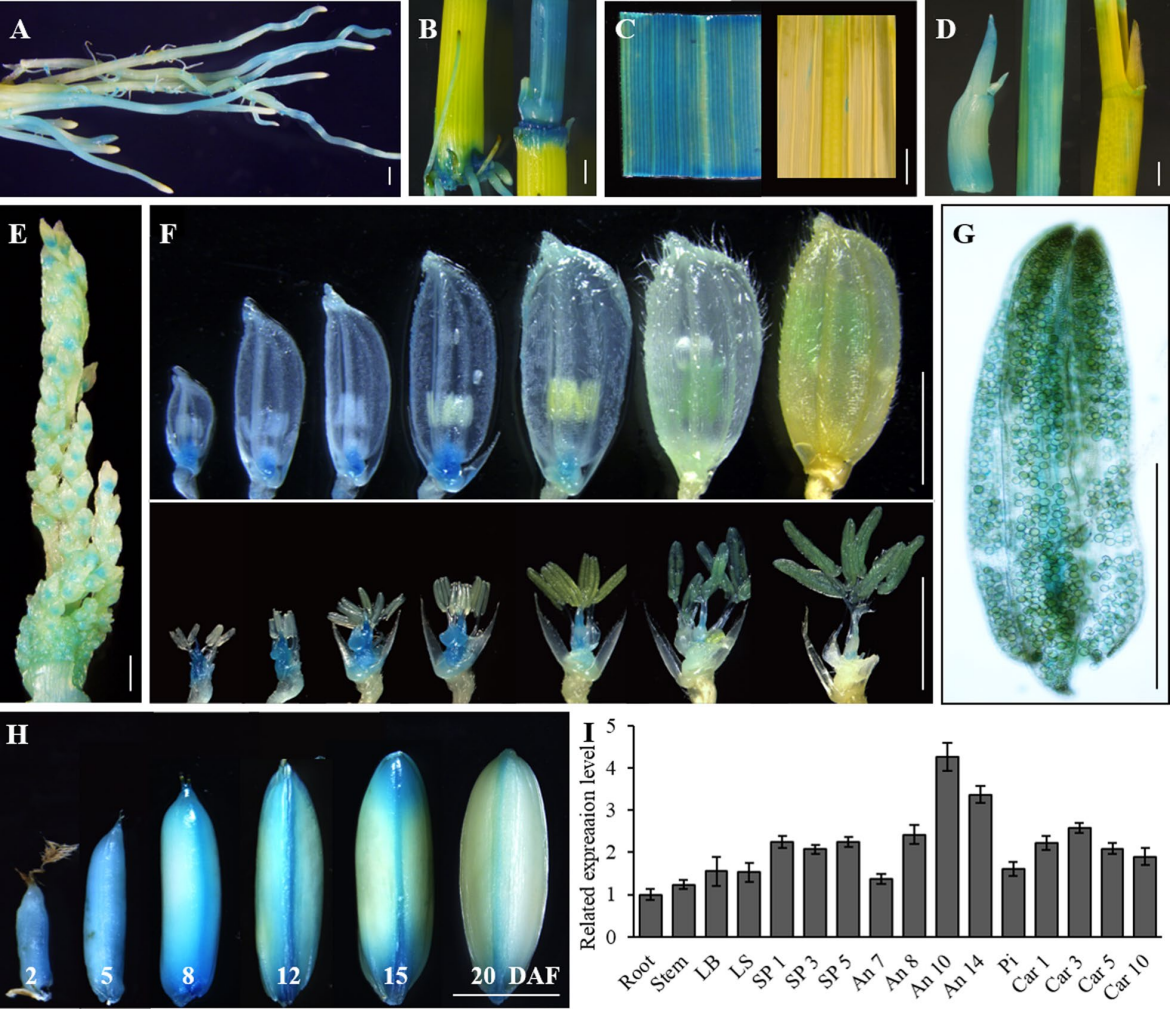
Expression pattern of *WS1* in rice. A-H *WS1* expression revealed by GUS staining. GUS activity was detected in the young root (**A**), developing culm (**B**), young leaf blade (**C**), young leaf sheath (**D**), developing spikelet and floral organs (**E**, **F**), maturing stamen and pollen (**G**), and developing caryopsis (**H**). I *WS1* expression detected by RT-qPCR analysis in different organs/tissues. The RT-qPCR analysis was performed with three biological replicates. The error bars indicated the standard deviation. DAP, days after pollination; LB, leaf blade; LS, leaf sheath; Pi, pistil; SP1, SP3, and SP5, young panicles of 1 cm, 3 cm, and 5 cm in length; An7, An8, An10, and An14, developing stamen at four different stages according to Zhang et al. (2011); Car1, Car 3, Car 5 and Car 10, developing caryopsis at four different days after pollination

*GUS* expression driven by the promoter of *WS1* indicated that *WS1* is ubiquitously expressed in various tissues. During the vegetative period, GUS activity was strong in the young or developing root, stem, leaf blade, and leaf sheath, but markedly weakened or even undetectable in maturing tissues (Fig. 5A–D). During the reproductive period, *WS1* was expressed in a young panicle (Fig. 5E). During spikelet formation, intense expression of *WS1* was mainly detected in developing pistils and receptacles, but very weak in lemma/palea and extra glume. *WS1* expression intensity gradually increased in the stamen as it developed, but the trend was the opposite in the pistil (Fig. 5F). In mature spikelets, *WS1* was expressed strongly in the stamen and pollen but weakly in the pistil, and almost none in other organs (Fig. 5F, G). *WS1* expression was high initially in developing caryopsis but significantly reduced in maturing seed (Fig. 5H). Interestingly, strong GUS activity was observed in the ovular vascular throughout caryopsis development (Fig. 5H). Consistent with GUS activity detection, RT-qPCR also indicated that *WS1* is ubiquitously expressed in various tissues or organs, with higher in reproductive organs and early caryopsis and the strongest in developing stamen (Fig. 5I). The rice genome contains ten predicted *GSL* genes. RT-qPCR analysis showed that compared with that during anther development, in the WT, *GSL3*, *GSL5*, and *GSL8* expression in the *ws1-1* was significantly higher than that in the WT, while the expression of the other six *GSL* genes did not change (Additional file 1: Fig. S6). This suggested that *GSL3*, *GSL5*, and *GSL8* might be partially redundant with *WS1* in function for microgametogenesis.

### *WS1* Affects the Deposition of Callose in Microspores

**Fig. 6:**
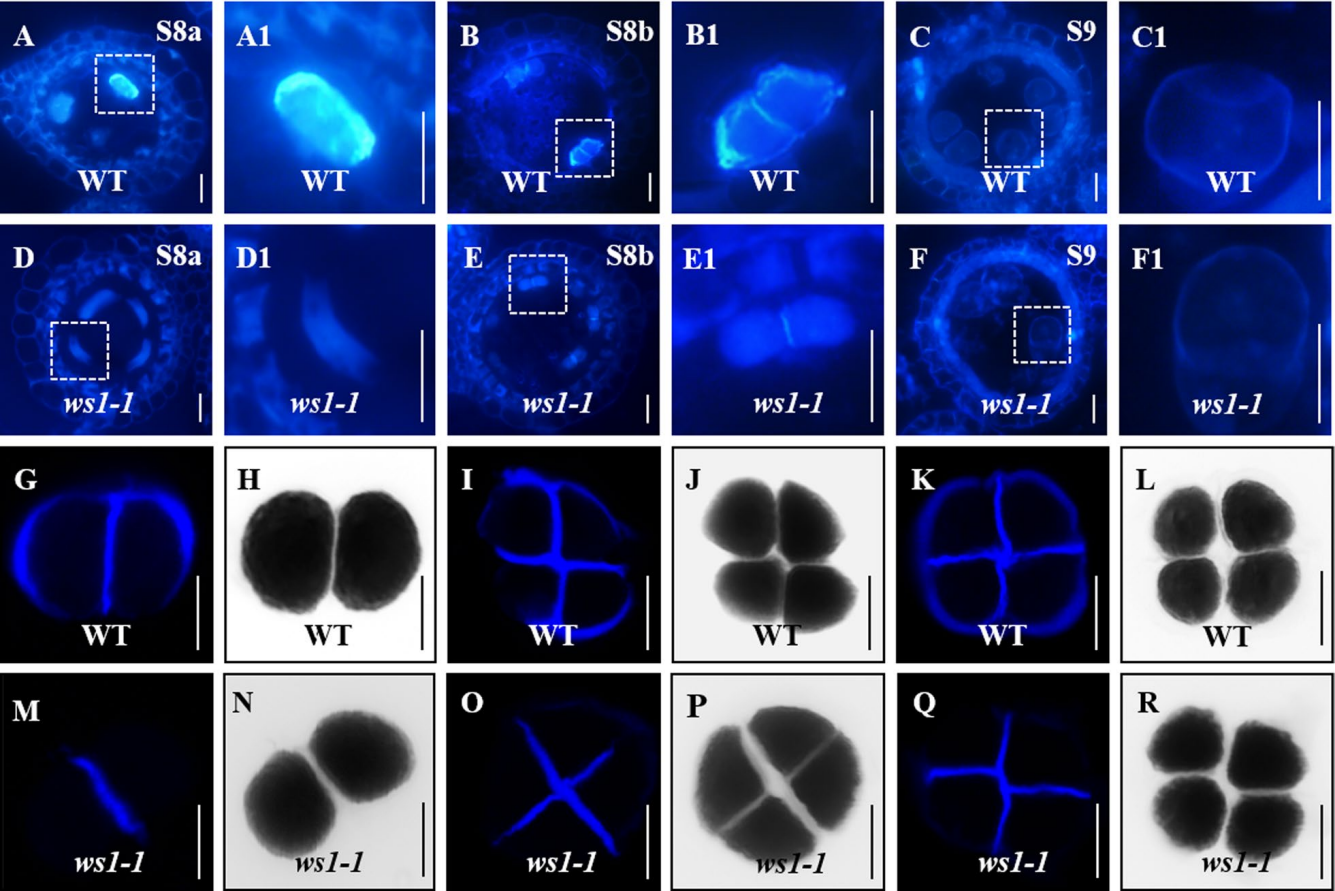
Loss of *WS1* function causes defective callose deposition during microsporogenesis. **A**–**F** Aniline blue stained anther sections of WT (**A**–**C**) and *ws1-1* (**D**–**F**) from stage 8a (S8a) to stage 9 (S9). **A1**–**F1** Magnified views of the boxed regions in (**A**–**F**) to show details. **G**–**R** Aniline blue-stained meiotic microspores in the WT (**G**–**L**) and *ws1-1* (**M**–**R**); staining of WT microspores in dyads (**G**, **H**) and tetrads (**I**–**L**); staining of *ws1-1* microspores of the dyad (**M**, **N**) and tetrad (**O**–**R**). Bright-field in (**H**, **J**, **L**, **N**, **P**, **R**). Bars = 10 μm

Considering that *WS1* was predicted to be a callose synthase gene and displayed an effect on microspore development from the dyad stage onwards (Fig. 2), we investigated its role in callose accumulation in microsporogenesis by aniline blue staining of the transverse sections of anthers. At stage 8, dyad and tetrad were formed with obvious callose deposition on the outer walls and in the cell plate of WT (Fig. 6A, B), but it appears that callose is gone at meiotic prophase I and very weak in dyad and tetrad (Fig. 6D, E). At stage 9, however, the released microspores in WT and *ws1-1* showed similar weak callose deposition (Fig. 6C, F). This suggested that *WS1* mainly affects callose deposition in the microspore of prophase I stage, dyad and tetrad. By direct staining of anthers, we obtained a clearer view of the callose deposition in dyad and tetrad. It could be seen that over 98% microspore cell in the dyad and tetrad was fully covered by callose with a high signal in the WT (Fig. 6G–L). In *ws1-1*, a strong callose signal was also observed in the cell plates of dyad and tetrad (though a little weaker than that in WT), but 78.9% of outer walls of dyad and tetrad had almost no callose (Fig. 6M–R), indicating that *WS1* is essential for callose deposition on the outer wall of microspore.

### *WS1* Impacts the Formation of an Annular Callose Sheath in Developing Caryopsis

**Fig. 7:**
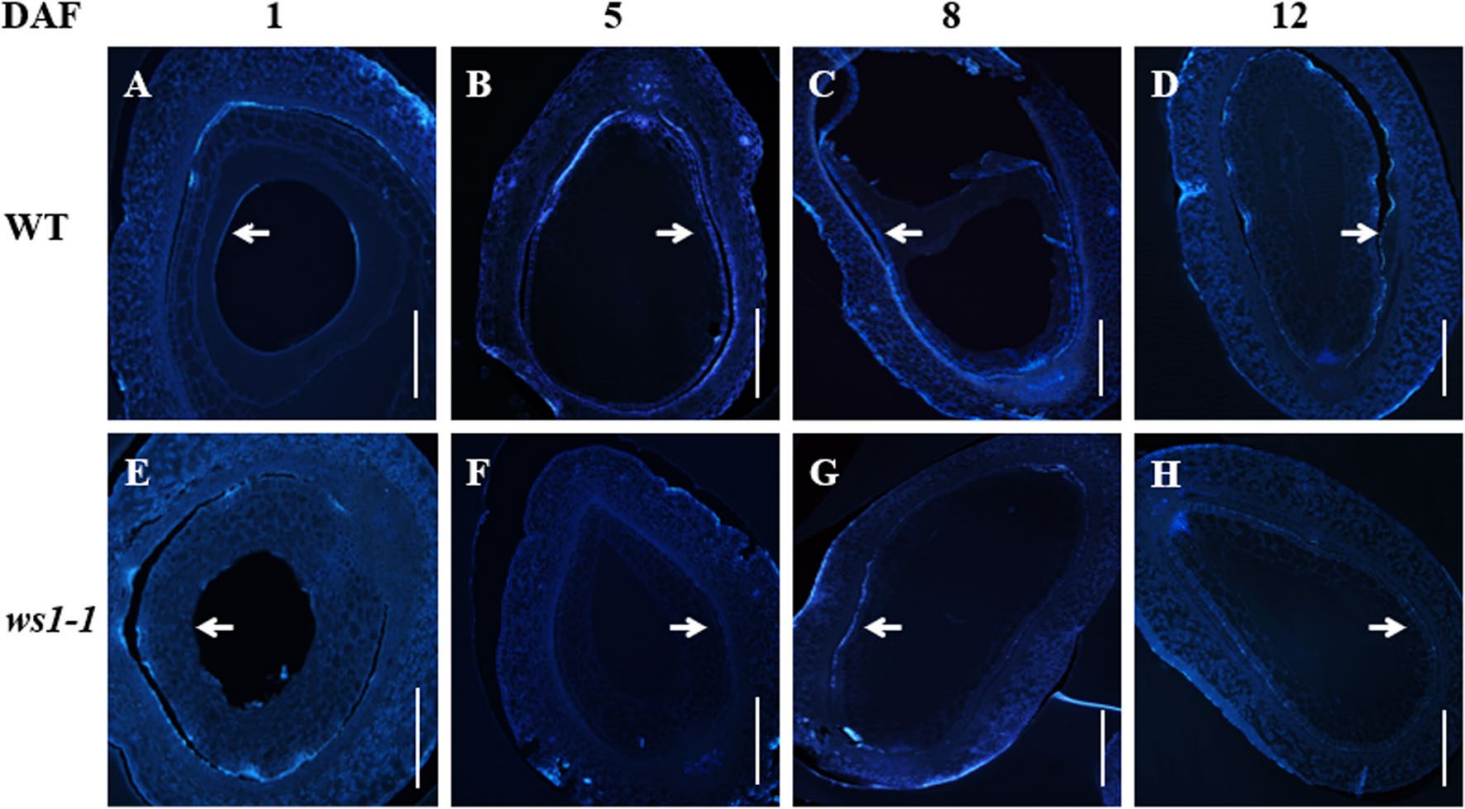
Absence of callose sheath during caryopsis development in *ws1-1*. **A**–**D** Aniline blue stained caryopsis sections of the WT at 1 (**A**), 5 (**B**), 8 (**C**), and 12 DAP (**D**). E–H Aniline blue stained caryopsis sections of *ws1-1* at 1 (**E**), 5 (**F**), 8 (**G**), and 12 DAP (**H**). Arrows indicate the zones of callose accumulation. Bars = 100 μm

The development of endosperm in rice requires a relatively independent environment. Previous studies showed that a sheath-like callose structure between the epidermis of the nucellar tissue and the internal integument is formed after pollination. The formation of this structure shows a dynamic change during the endosperm development and may be closely related to the regulation of water and nutrients during the development of the embryo and endosperm (Wang et al. 2004). By staining the cross-sections of developing caryopses with aniline blue, we observed a similar phenomenon in the WT. Callose was deposited between the epidermis of the nucellar tissue and the internal integument at 1 DAP, forming an almost closed callose sheath (Fig. 7A). The sheath was kept for at least 10 days (Fig. 7B, C). Then, it began to be gradually degraded from 12 DAP (Fig. 7D) and finally completely disappeared in the mature seed. Compared with the WT, *ws1-1* had much less callose deposition in the developing caryopsis, which resulted in very weak and discontinuous callose zones and therefore could not form a close callose sheath to provide an independent environment for the development of endosperm (Fig. 7E–H). This might be one of the main reasons for the endosperm abortion in *ws1-1*.

## Discussions

It has been known that *GSL* genes are indispensable for some important highly specialized developmental and cellular processes involving callose synthesis and deposition in plants. At present, the best-characterized functions of *GSL* genes are those for pollen formation (Chen et al. 2009; Song et al. 2016). Knowledge about the roles of *GSL* genes in seed development is still very limited. In this study, we identified and characterized a *GSL* gene named *WS1*/*OsGSL2* in rice. Although the expression of *OsGS2* is ubiquitous, the aberrant phenotypes only manifest during reproductive development, indicating that *OsGSL2* has an auxiliary role in other organs, likely due to its low expression and the redundancy effect of other GSL genes in these organs. Our results indicated that *OsGSL2* is crucial not only for pollen formation but also for embryo and endosperm development.

### The Role of *OsGSL* Genes in Pollen Development in Rice

As a family with 12 members in *Arabidopsis*, multiple *GSL* genes have been found to be involved in pollen development. *AtGSL1* and *AtGSL5* are requisite for separating tetrad microspores and preventing microspore degeneration (Enns et al. 2005). *AtGSL2* plays a role in the cell wall formation of mother cells and in the patterning of pollen exine (Dong et al. 2005). *AtGSL8* and *AtGSL10* are independently required for the asymmetric division of microspores and for the entry of microspores into mitosis (Töller et al. 2008; Huang et al. 2009). Mutation of these genes often leads to pollen abortion and low fertility.

In rice, only one *GSL* gene (*OsGSL5*, the homolog of *AtGSL2*) was previously found to play a vital role in pollen development (Shi et al. 2015)*. OsGSL5* is expressed in most plant tissues but is highest in developing and mature pollens. Knockout or knockdown of *OsGSL5* causes defective callose deposition on the meiocyte cell wall and dyad/tetrad cell plates and therefore produces collapsed pollen grains without callose wall, leading to a severe reduction of pollen fertility (Shi et al 2015). A recent study (Somashekar et al. 2023) highlighted that the callose deposition is mediated by GSL5 during meiosis stages, in addition to the pollen stage (Shi et al. 2015). In this study, we revealed that *OsGSL2* also affected pollen fertility. *OsGSL2* is closely related to *AtGSL1* and *AtGSL5*. Similar to *OsGSL5*, *OsGSL2* was also expressed in various tissues, with the highest expression in developing anther and pollen (Fig. 5I). Disrupting *OsGSL2* also reduced callose deposition in dyad and tetrad, resulting in pollen abortion and a decrease in fertile pollens (Figs. 2, 6). However, the function of *OsGSL2* is not exactly the same as that of *OsGSL5*. The most apparent difference between the two genes is that while loss of *OsGSL5* function can significantly reduce the callose deposition on cell plates (Shi et al. 2015), mutation of *OsGSL2* showed little influence on cell plates (Fig. 6). This was consistent with the cytological observation that dyad and tetrad microspores could be successfully produced in *ws1-1* by meiosis (Fig. 2), which requires accurate callose deposition on the cell plate. Moreover, it appears that callose is gone at meiotic prophase I of *ws1-1* (Fig. 6D), consistent with the *Osgsl5* phenotype (Somashekar et al. 2023), indicating that *ws1-1* shows more severe callose accumulation defects during meiosis than at dyad and tetrad. Furthermore, *ws1-1* likely shows more severe callose accumulation defects than *Osgsl5*, which explains about why callose is greatly reduced in *ws1-1* in spite of the upregulation of *GSL5* (Additional file 1, Fig. S9). Together, these results inferred that *OsGSL2* and *OsGSL5* must be partially redundant in function because callose does not completely disappear during microsporogenesis and there is still a small proportion of fertile pollens produced in either single mutant of them.

Apart from *OsGSL2* and *OsGSL5*, most of the other *OsGSL* genes are also expressed in developing anthers (Yamaguchi et al. 2006). It was found that knockout of *OsGSL5* can increase the expression of *OsGSL1*, *OsGSL2*, *OsGSL4*, *OsGSL8*, *OsGSL9*, and *OsGSL10* (Shi et al. 2015), and disruption of *OsGSL2* could enhance the expression of *OsGSL3*, *OsGSL5*, and *OsGSL8* (Additional file 1: Fig. S9). This implies that some of the other *OsGSL* genes might probably also play a role in pollen development. Nonetheless, it has been confirmed that *OsGSL8* does not affect pollen fertility, although its loss-of-function mutation results in smaller anthers (Song et al. 2016).

### The Role of *OsGSL* Genes in Seed Development in Rice

Seed development starts from the completion of double fertilization. Ovules are female reproductive organs of angiosperms, containing sporophytic integuments and gametophytic embryo sacs. After fertilization, the embryo sac develops into the embryo and endosperm, and integument into the seed coat, whereas other nucellus cells undergo a degenerative process known as programmed cell death (Krishnan and Dayanandan 2003; Hands et al. 2016; Wu et al. 2016a). In cereal crops, the endosperm is the main component of grain, and embryogenesis is dependent upon the accompanying endosperm development. The early development of endosperm comprises several short but critical events, including the formation and division of the primary endosperm nucleus, positional distribution of free nuclei, and endosperm cellularization (Hands et al. 2016), which largely determine seed size and quality (Olsen 2004; Garcia et al. 2005; Dante et al. 2014).

One *OsGSL* gene (*OsGSL8/CRR1*) has been identified to affect seed development in rice before (Song et al. 2016). It is found that *OsGSL8* regulates the vascular cell patterning in ovaries and receptacles. Loss of *OsGSL8* function makes ovary expansion arrested due to less efficient unloading of carbohydrates from the lateral vasculature into the developing caryopsis, resulting in smaller grains. Compared with *OsGSL8*, it is obvious that *OsGSL2* functions quite differently in seed development. *OsGSL2* is essential for embryo differentiation and endosperm-free nucleus positioning. It plays a distinct role in seed initiation and filling.

Embryo and endosperm development is a very complicated biological process, in which many genes must be involved. Apart from the above two *GSL* genes, several other genes showing similar functions in early seed development have been identified in rice. Knock-down or knock-out of these genes caused partially or totally similar cellular defects and seed dephenotypes to that of *ws1-1* (Fig. 1). Rice *LEAF AND FLOWER*-related gene (*OsLFR*), which encodes an interaction partner of SWITCH/SUCROSE NON-FERMENTABLE (SWI/SNF) in the ATP-dependent chromatin-remodeling complex, is essential for early endosperm and embryo development (Qi et al. 2020). In *Oslfr*, the embryo is reduced in size and fails to differentiate, the endosperm has fewer free nuclei and the cellularization is arrested. Maternally expressed polycomb group gene *OsEMF2a* promotes endosperm cellularization, which is associated with an unusual activation of type I MADS-box genes through OsEMF2a-PRC2 (Polycomb Repressive Complex 2)-mediated H3K27me3. *Osemf2a* delays the cellularization of endosperm cells and halts embryo development (Cheng et al. 2021). By contrast, *OsMADS78* and *OsMADS79* inhibit endosperm cellularization. Overexpressing *OsMADS78* or *OsMADS79* causes delayed endosperm cellularization, while knockout of either of them leads to precocious endosperm cellularization, and no viable seeds can be produced when the two genes are disrupted simultaneously (Paul et al. 2020). *OsMADS29* regulates nucellus degradation after fertilization by directly promoting the expression of Cys protease and programmed cell death-related genes (Yin and Xue 2012). Suppression of *OsMADS29* results in shrunken seeds due to the defective degradation of the nucellus, similar to the phenomenon observed in *ws1-1* (Fig. 3M, N, P, Q).

Although seed development normally initiates from double fertilization, abnormal seed development independent of fertilization has also been observed. In *Arabidopsis*, several studies showed that pollen tube contents can initiate ovule enlargement and enhance seed coat development without fertilization (Kasahara et al. 2016; Zhong et al. 2017; Liu et al. 2019). In rice, Knockout of the *GENERATIVE CELL SPECIFIC 1* (*OsGCL1*) gene leads to fertilization failure and pollen tube-dependent ovule enlargement, producing seeds similar to those of *ws1-1* (Honma et al. 2020). The dioxygenase for auxin oxidation (*DAO*) gene, encoding a putative 2-oxoglutarate-dependent-Fe (II) dioxygenase and catalyzing the conversion of IAA into OxIAA, also causes fertilization-independent abnormal seed development in rice when its function is lost. Similar to the *ws1-1* seed, the unfertilized seed in the *dao* mutant is filled with sucrose-rich liquid without starch accumulation (Zhao et al. 2013).

In short, many critical genes are involved in seed development in rice. Disruption of these genes may also result in embryo abortion and endosperm loss similar to *ws1-1* owing to various causes, including a stop for reduction of endosperm nuclear production, arrest of cellularization, failure of endosperm free nucleus migration and embryo differentiation, and premature or delayed degeneration of nucellus cells. These genes together with *OsGSL2* may form a complex regulatory network for rice seed development. More studies are needed to explore the network.

## Conclusions

*WS1*/*OsGSL2* is highly expressed in reproductive organs and developing seeds and is essential for rice fertility and is required for embryo differentiation and endosperm-free nucleus positioning. Disrupting *OsGSL2* reduced callose deposition in meiotic microspores and impaired the formation of annular callose sheath in developing caryopsis, leading to defective pollen and watery seed lacking embryo and endosperm. These findings indicate a distinct role of the *GSL* gene in seed initiation, which provides new insight into the regulation of seed development in cereals.

## Methods

### Plant Materials

Two rice cultivars, Nipponbare (*japonica*) and Lemont (*indica*), and five mutant lines, *ws1-1* to *ws1-5*, were used in this study. The mutant *ws1-1* was obtained from Nipponbare by EMS mutagenesis, while *ws1-2* to *ws1-5* were generated from Nipponbare by editing the target gene *WS1* using the CRISPR/Cas9 system.

### Identification of *WS1* Gene

Primary mapping of the *WS1* gene was conducted by linkage analysis based on 163 mutant plants selected from the F_2_ generation of *ws1-1* × Lemont. InDel markers developed in this study as well as the available RM-series SSR markers were used for the linkage analysis (Additional file 2: Table S1). To narrow down the interval of *WS1* identified by the linkage analysis, next-generation sequencing-based bulked segregant analysis (BSA-seq) was performed using the F_3_ generation. The fresh leaves of equal amounts from 40 homozygous wild-types (WT) F_2:3_ lines and those from 40 mutant F_2:3_ lines were mixed respectively to make a pair of WT and mutant DNA pools. The two DNA pools together with the genomic DNA of *ws1-1* were sent to Xiamen Jointgene Technologies Corporation for deep sequencing using the Illumina PE Genome Analyzer. Based on the sequencing data of the two pools and using the Nipponbare genome as a reference genome (http://rice.plantbiology.msu.edu), the genomic region harboring *WS1* was identified. According to the gene mapping results, the candidate gene of *WS1* was identified by comparing the sequence of the estimated *WS1* region in *ws1-1* with the corresponding sequence in Nipponbare.

### Vector Construction and Plant Transformation

For the genetic complementation test of *OsGSL2* (the candidate gene of *WS1*), a 7764-bp genomic DNA sequence covering the promoter, gene, and downstream region of *OsGSL2* was amplified from Nipponbare, and inserted into the binary vector pCAMBIA1300. For the knockout of *OsGSL2*, the CRISPR/Cas9 system was used. The CRISPR targets for *OsGSL2* were selected as described by Miao et al. (2013), and the vector construction was performed according to the manufacturer’s instructions for the regent kit (VIEWSOLID Biotech, China). For display of the expression pattern of *OsGSL2*, a 3000-bp promoter sequence of *OsGSL2* was amplified from Nipponbare and fused into the GUS reporter gene in pCAMBIA1391Z. The primers used for vector construction are listed in (Additional file 2: Table S1). All constructs were introduced into *Agrobacterium tumefaciens* strain EHA105 and transferred into rice. The vector for the complementation test, the vectors for CRISPR/Cas9, and the vector for GUS expression were transferred into *ws1-1*, Nipponbare, and ZH11, respectively. The genomic region surrounding the CRISPR target sites for *OsGSL2* was amplified and sequenced to identify mutants. Histochemical assay for GUS activity in transgenic plants was performed as described (Jefferson et al. 1987).

### Scanning Electron Microscopy (SEM)

SEM observation of anthers was performed according to Duan et al. (2019). The mature anthers were fixed in 2.5% glutaric dialdehyde and washed with a sodium phosphate buffer (0.1 M, pH7.0); further fixed in 1% osmic acid (w/v) for 1–2 h and again washed with the sodium phosphate buffer; dehydrated with an ethanol series, incubated in ethanol-tert butanol and then in tert butanol; and finally observed with a TM3030 Plus scanning electronic microscope (Hitachi, Japan).

### Histological Observations

Observation of pollen development was performed by resin slicing as described (Duan et al. 2019). Rice anthers at various stages were fixed in 2.5% glutaraldehyde solution, dehydrated using a graded ethanol series (20, 40, 60, 80, 100, 100%), and embedded in Leica 7022 historesin with 1/16 volume of Hardner (Leica, Nussloch, Germany). Samples were sectioned to 4 μm, stained with 0.1% Toluidine Blue-O (Sigma, St. Louis, MO, USA), and observed under a Leica DM 4000B light microscope.

Pollen tube growth was observed by aniline blue staining as described (Li et al. 2013). Pollinated pistils of the mutant and WT were collected at different times and fixed in Carnoy’s fixing reagent (30% chloroform, 10% acetic acid, and 57% ethanol). The samples were washed with water, incubated in 10 mol l^−1^ NaOH at 56℃ for 6–10 min, stained in 0.1% aniline blue solution for 8–12 h, and observed under a Leica DM 4000B light microscope.

Observation of embryo sac and seed development was performed by whole-mount eosin B-staining as described (Huang et al. 2017). Ovaries at various stages were collected carefully, fixed in FAA overnight, washed with 50% ethanol, and stored in 70% ethanol at 4 °C. The samples were scanned under a Leica SP8 laser scanning confocal microscope. The excitation wavelength was 543 nm, and the emission wavelengths were 550–630 nm. Images of the ovaries were recorded using the software for the microscope.

### RNA Isolation and RT-qPCR Analysis

RT-qPCR analysis was performed according to Duan et al. (2019). Briefly, Total RNA was isolated using a Trizol reagent kit (Omega R6934, USA). Reverse transcription was performed using the PrimeScript™ RT reagent kit (Takara RR047A, China). qPCR analysis was performed with three biological replicates using the SYBR *Premix Ex Taq* II (Takara, China) in an ABI QuantStudio 3 detect system. Amplification of *Actin* was used as an internal control to normalize all data. Primers used for RT-qPCR analysis are listed in (Additional file 2: Table S2).

### Callose Staining with Aniline Blue

Pollen staining was performed according to Shi et al. (2015). First, anthers were processed through an ethanol series (70, 50, 30, and 15%) and then transferred to distilled water. Next, the anthers were placed on a glass slide and squeezed with tweezers to release pollens. Finally, the pollens were stained with 0.01% (w/v) aniline blue solution. Staining of the transverse sections of anthers and developing seeds was performed according to Wang et al. (2020). The sections were stained for 10 min at room temperature with 0.01% (w/v) aniline blue solution and washed with phosphate buffer. The samples were visualized under UV light with a confocal laser scanning microscope (LEICA-SP8).

### Subcellular Localization

In-frame OsGSL2-GFP fusion protein driven by the 35S promoter and p35S::PIP2:DsRed (Plasma membrane marker) were cotransformed into rice protoplasts by PEG. Then, the protoplasts were observed with a confocal laser scanning microscope (LEICA-SP8). The primers for amplifying *OsGSL2* cDNA are listed in (Additional file 2: Table S2).

### Supplementary Information

**Additional file 1: Fig. S1** Plants of wild-type and *ws1-1*. **Fig. S2** Sugar content of the fluid obtained from *ws1-1* caryopses at 10 days after pollination. **Fig. S3** Compares the developing caryopses from WT and *ws1-1*. **Fig. S4** Results of artificial fertilization within or between WT and *ws1-1*. **Fig. S5** Comparison of microsporogenesis at stages 6 and 7 in WT and mutant anthers*.* **Fig. S6** The phenotypes of the seeds, anthers and pollens, and the early seed development in the complementary and knockout plants. **Fig. S7** The predicted protein structure of WS1/OsGSL2. **Fig. S8** WS1 is the homolog of AtGSL1 and AtGSL5. **Fig. S9** qRT-PCR analysis of *OsGSL* genes in the WT and *ws1-1* anthers at microspore development.

**Additional file 2: Table S1** Primers used in this study. **Table S2** Results of *WS1* sequence editing by Cas9-induced knockout.

## Data Availability

All data supporting the findings of this study are available from the corresponding author on reasonable request.

## References

[CR1] Albert B, Ressayre A, Nadot S (2011). Correlation between pollen aperture pattern and callose deposition in late tetrad stage in three species producing atypical pollen grains. Am J Bot.

[CR2] Barratt DH, Kölling K, Graf A, Pike M, Calder G, Findlay K, Zeeman SC, Smith AM (2011). Callose synthase GSL7 is necessary for normal phloem transport and inflorescence growth in Arabidopsis. Plant Physiol.

[CR3] Chen XY, Kim JY (2009). Callose synthesis in higher plants. Plant Signal Behav.

[CR4] Chen XY, Liu L, Lee E, Han X, Rim Y, Chu H, Kim S-W, Sack F, Kim J-Y (2009). The Arabidopsis callose synthase gene *GSL8* is required for cytokinesis and cell patterning. Plant Physiol.

[CR5] Cheng X, Pan M, Zhiguo E, Zhou Y, Niu B, Chen C (2021). The maternally expressed polycomb group gene *OsEMF2a* is essential for endosperm cellularization and imprinting in rice. Plant Commun.

[CR6] Dante RA, Larkin BA, Sabelli PA (2014). Cell cycle control and seed development. Front Plant Sci.

[CR7] Dong X, Hong Z, Sivaramakrishnan M, Mahfouz M, Verma DP (2005). Callose synthase (CalS5) is required for exine formation during microgametogenesis and for pollen viability in *Arabidopsis*. Plant J.

[CR8] Duan Y, Chen Y, Li W, Pan M, Qu X, Shi X, Cai Z, Liu H, Zhao F, Kong L, Ye Y, Wang F, Xue Y, Wu W (2019). *RETINOBLASTOMA-RELATED* genes specifically control inner floral organ morphogenesis and pollen development in rice. Plant Physiol.

[CR9] Enns LC, Kanaoka MM, Torii KU, Comai L, Okada K, Cleland RE (2005). Two callose synthases, GSL1 and GSL5, play an essential and redundant role in plant and pollen development and in fertility. Plant Mol Biol.

[CR10] Franklin-Tong VE (1999). Signaling and the modulation of pollen tube growth. Plant Cell.

[CR11] Garcia D, Fitz Gerald JN, Berger F (2005). Maternal control of integument cell elongation and zygotic control of endosperm growth are coordinated to determine seed size in *Arabidopsis*. Plant Cell.

[CR12] Guseman JM, Lee JS, Bogenschutz NL, Peterson KM, Virata RE, Xie B, Kanaoka MM, Hong Z, Torii KU (2010). Dysregulation of cell-to-cell connectivity and stomatal patterning by loss-of-function mutation in *Arabidopsis* chorus (glucan synthase-like 8). Development.

[CR13] Han X, Hyun TK, Zhang M, Kumar R, Koh EJ, Kang BH, Lucas WJ, Kim JY (2014). Auxin-callose-mediated plasmodesmal gating is essential for tropic auxin gradient formation and signaling. Dev Cell.

[CR14] Hands P, Rabiger DS, Koltunow A (2016). Mechanisms of endosperm initiation. Plant Reprod.

[CR15] Hong Z, Delauney AJ, Verma DP (2001). A cell plate-specific callose synthase and its interaction with phragmoplastin. Plant Cell.

[CR16] Honma Y, Adhikari PB, Kuwata K, Kagenishi T, Yokawa K, Notaguchi M, Kurotani K, Toda E, Bessho-Uehara K, Liu X, Zhu X, Wu X, Kasahara RD (2020). High-quality sugar production by *osgcs1* rice. Commun Biol.

[CR17] Huang L, Chen XY, Rim Y, Han X, Cho WK, Kim SW, Kim JY (2009). *Arabidopsis* glucan synthase-like 10 functions in male gametogenesis. J Plant Physiol.

[CR18] Huang X, Peng X, Sun MX (2017). *OsGCD1* is essential for rice fertility and required for embryo dorsal-ventral pattern formation and endosperm development. New Phytol.

[CR19] Janas AB, Marciniuk J, Szeląg Z, Musiał K (2022). New facts about callose events in the young ovules of some sexual and apomictic species of the Asteraceae family. Protoplasma.

[CR20] Jefferson RA, Kavanagh TA, Bevan MW (1987). GUS fusions: beta-glucuronidase as a sensitive and versatile gene fusion marker in higher plants. EMBO J.

[CR21] Kasahara RD, Notaguchi M, Nagahara S, Suzuki T, Susaki D, Honma Y, Maruyama D, Higashiyama T (2016). Pollen tube contents initiate ovule enlargement and enhance seed coat development without fertilization. Sci Adv.

[CR22] Krishnan S, Dayanandan P (2003). Structural and histochemical studies on grain-filling in the caryopsis of rice (*Oryza sativa* L.). J Biosci.

[CR23] Li S, Li W, Huang B, Cao X, Zhou X, Ye S, Li C, Gao F, Zou T, Xie K, Ren Y, Ai P, Tang Y, Li X, Deng Q, Wang S, Zheng A, Zhu J, Liu H, Wang L, Li P (2013). Natural variation in *PTB1* regulates rice seed setting rate by controlling pollen tube growth. Nat Commun.

[CR24] Liu HZ, Zhang GS, Zhu WW, Ba QS, Niu N, Wang JW, Ma SC, Wang JS (2015). Relationship between male sterility and β-1,3-glucanase activity and callose deposition-related gene expression in wheat (Triticum aestivum L.). Genet Mol Res.

[CR25] Liu X, Adhikari PB, Kasahara RD (2019). Pollen tube contents from failed fertilization contribute to seed coat initiation in *Arabidopsis*. F1000Res.

[CR26] Miao J, Guo D, Zhang J, Huang Q, Qin G, Zhan X, Wan J, Gu H, Qu LJ (2013). Targeted mutagenesis in rice using CRISPR-Cas system. Cell Res.

[CR27] Nedukha OM (2015). Callose: localization, functions, and synthesis in plant cells. Cytol Genet.

[CR28] Nishikawa S, Zinkl GM, Swanson RJ, Maruyama D, Preuss D (2005). Callose (beta-1,3 glucan) is essential for Arabidopsis pollen wall patterning, but not tube growth. BMC Plant Biol.

[CR29] Nowack MK, Grini PE, Jakoby MJ, Lafos M, Koncz C, Schnittger A (2006). A positive signal from the fertilization of the egg cell sets off endosperm proliferation in angiosperm embryogenesis. Nat Genet.

[CR30] Olsen OA, Becraft PW (2013) Endosperm development. In: Seed genomics, pp 43–62

[CR31] Olsen OA (2004). Nuclear endosperm development in cereals and *Arabidopsis thaliana*. Plant Cell.

[CR32] Olsen OA (2020). The modular control of cereal endosperm development. Trends Plant Sci.

[CR33] Paul P, Dhatt BK, Miller M, Folsom JJ, Wang Z, Krassovskaya I, Liu K, Sandhu J, Yu H, Zhang C, Obata T, Staswick WH (2020). *MADS78* and *MADS79* are essential regulators of early seed development in rice. Plant Physiol.

[CR34] Qi D, Wen Q, Meng Z, Yuan S, Guo H, Zhao H, Cui S (2020). *OsLFR* is essential for early endosperm and embryo development by interacting with SWI/SNF complex members in *Oryza sativa*. Plant J.

[CR35] Qin P, Ting D, Shieh A, McCormick S (2012). Callose plug deposition patterns vary in pollen tubes of Arabidopsis thaliana ecotypes and tomato species. BMC Plant Biol.

[CR36] Sabelli PA, Larkins BA (2009). The development of endosperm in grasses. Plant Physiol.

[CR37] Shi X, Han X, Lu TG (2016). Callose synthesis during reproductive development in monocotyledonous and dicotyledonous plants. Plant Signal Behav.

[CR38] Shi X, Sun X, Zhan Z, Feng D, Zhang Q, Han L, Wu J, Lu T (2015). GLUCAN SYNTHASE-LIKE 5 (GSL5) plays an essential role in male fertility by regulating callose metabolism during microsporogenesis in rice. Plant Cell Physiol.

[CR39] Slewinski TL, Baker RF, Stubert A, Braun DM (2012). Tie-dyed2 encodes a callose synthase that functions in vein development and affects symplastic trafficking within the phloem of maize leaves. Plant Physiol.

[CR40] Somashekar H, Mimura M, Tsuda K, Nonomura KI (2023). Rice GLUCAN SYNTHASE-LIKE5 promotes anther callose deposition to maintain meiosis initiation and progression. Plant Physiol.

[CR41] Song L, Wang R, Zhang L, Wang Y, Yao S (2016). CRR1 encoding callose synthase functions in ovary expansion by affecting vascular cell patterning in rice. Plant J.

[CR42] Thiele K, Wanner G, Kindzierski V, Jürgens G, Mayer U, Pachl F, Assaad FF (2009). The timely deposition of callose is essential for cytokinesis in *Arabidopsis*. Plant J.

[CR43] Töller A, Brownfield L, Neu C, Twell D, Schulze-Lefert P (2008). Dual function of Arabidopsis glucan synthase-like genes GSL8 and GSL10 in male gametophyte development and plant growth. Plant J.

[CR44] Verma DPS, Hong Z (2001). Plant callose synthase complexes. Plant Mol Bio.

[CR45] Wang B, Andargie M, Fang R (2022). The function and biosynthesis of callose in high plants. Heliyon.

[CR46] Wang B, Fang R, Zhang J, Han J, Chen F, He F, Liu Y, Chen L (2020). Rice LecRK5 phosphorylates a UGPase to regulate callose biosynthesis during pollen development. J Exp Bot.

[CR47] Wang L, Liu X, Lu Y, Feng J, Xu X, Xu S (2004). Endosperm development in autotetraploid rice: The development of the cellulose wall of aleuronic layer cell, starch accumulation of endosperm and formation of a callose “sheath-like” structure. Zhongguo Shuidao Kexue.

[CR48] Wu X, Liu J, Li D, Liu CM (2016). Rice caryopsis development I: Dynamic changes in different cell layers. J Integr Plant Biol.

[CR49] Wu X, Liu J, Li D, Liu CM (2016). Rice caryopsis development II: Dynamic changes in the endosperm. J Integr Plant Biol.

[CR50] Yamaguchi T, Hayashi T, Nakayama K, Koike S (2006). Expression analysis of genes for callose synthases and Rho-type small GTP-binding proteins that are related to callose synthesis in rice anther. Biosci Biotechnol Biochem.

[CR51] Yin LL, Xue HW (2012). The MADS29 transcription factor regulates the degradation of the nucellus and the nucellar projection during rice seed development. Plant Cell.

[CR52] Zavaliev R, Ueki S, Epel BL, Citovsky V (2011). Biology of callose (b-1,3-glucan) turnover at plasmodesmata. Protoplasma.

[CR53] Zhang D, Luo X, Zhu L (2011). Cytological analysis and genetic control of rice anther development. J Genet Genomics.

[CR54] Zhang C, Shen Y, Tang D, Shi W, Zhang D, Du G, Zhou Y, Liang G, Li Y, Cheng Z (2018). The zinc finger protein DCM1 is required for male meiotic cytokinesis by preserving callose in rice. PLoS Genet.

[CR55] Zhao Z, Zhang Y, Liu X, Zhang X, Liu S, Yu X, Ren Y, Zheng X, Zhou K, Jiang L, Guo X, Gai Y, Wu C, Zhai H, Wang H, Wan J (2013). A Role for a Dioxygenase in Auxin Metabolism and Reproductive Development in Rice. Dev Cell.

[CR56] Zheng Y, Wang Z (2015). The cereal starch endosperm development and its relationship with other endosperm tissues and embryo. Protoplasma.

[CR57] Zhong S, Zhang J, Qu LJ (2017). The signals to trigger the initiation of ovule enlargement are from the pollen tubes: The direct evidence. J Integr Plant Biol.

