# Supplementary material for: Glucan Synthase-like 2 is Required for Seed Initiation and Filling as Well as Pollen Fertility in Rice
Source: Rice (N Y). 2023 Oct 7;16:44. doi: 10.1186/s12284-023-00662-z (PMC10560172; doi:10.1186/s12284-023-00662-z)
Supplement: Supplementary file 1 — Additional file 1: Fig. S1 Plants of wild-type and ws1-1. Fig. S2 Sugar content of the fluid obtained from ws1-1 caryopses at 10 days after pollination. Fig. S3 Compares the developing caryopses from WT and ws1-1. Fig. S4 Results of artificial fertilization within or between WT and ws1-1. Fig. S5 Comparison of microsporogenesis at stages 6 and 7 in WT and mutant anthers. Fig. S6 The phenotypes of the seeds, anthers and pollens, and the early seed development in the complementary and knockout plants. Fig. S7 The predicted protein structure of WS1/OsGSL2. Fig. S8 WS1 is the homolog of AtGSL1 and AtGSL5. Fig. S9 qRT-PCR analysis of OsGSL genes in the WT and ws1-1 anthers at microspore development. [file 12284_2023_662_MOESM1_ESM.docx]

**Additional file 1**


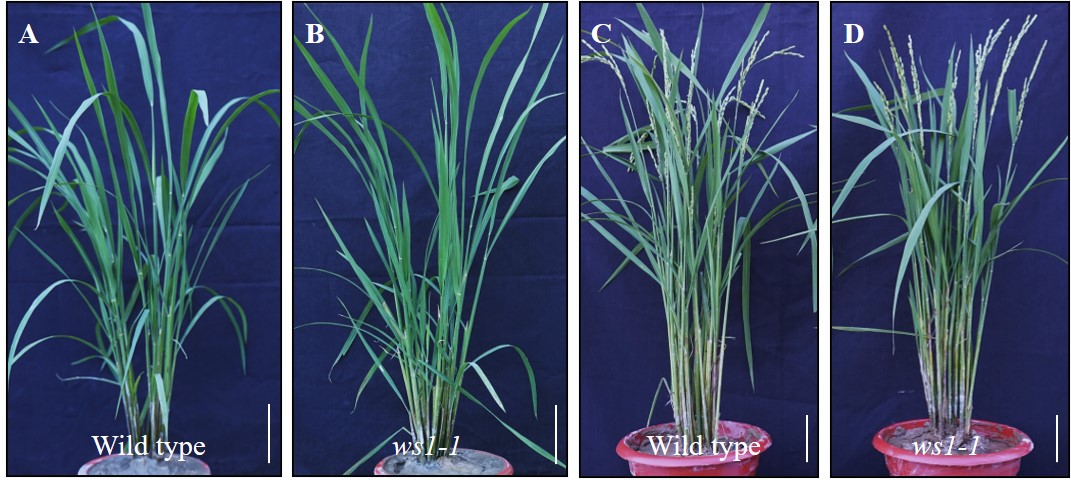


**Fig. S1 Plants of the wild type and *ws1-1*.**

No significant morphological difference was observed between the wild type and *ws1-1* at the late tillering stage (A, B) and flowering stage (C, D). Bar = 10 cm.

**
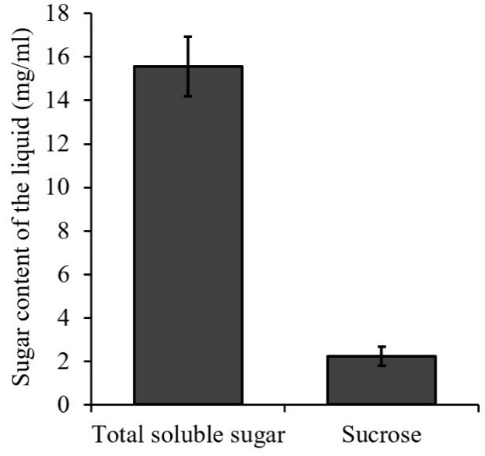
**

**Fig. S2 Sugar content in the liquid in *ws1-1* caryopses at 10 days after pollination.**

The total soluble sugar included sucrose, fructose, and glucose. The error bars indicate the standard deviations of three biological replicates.

**
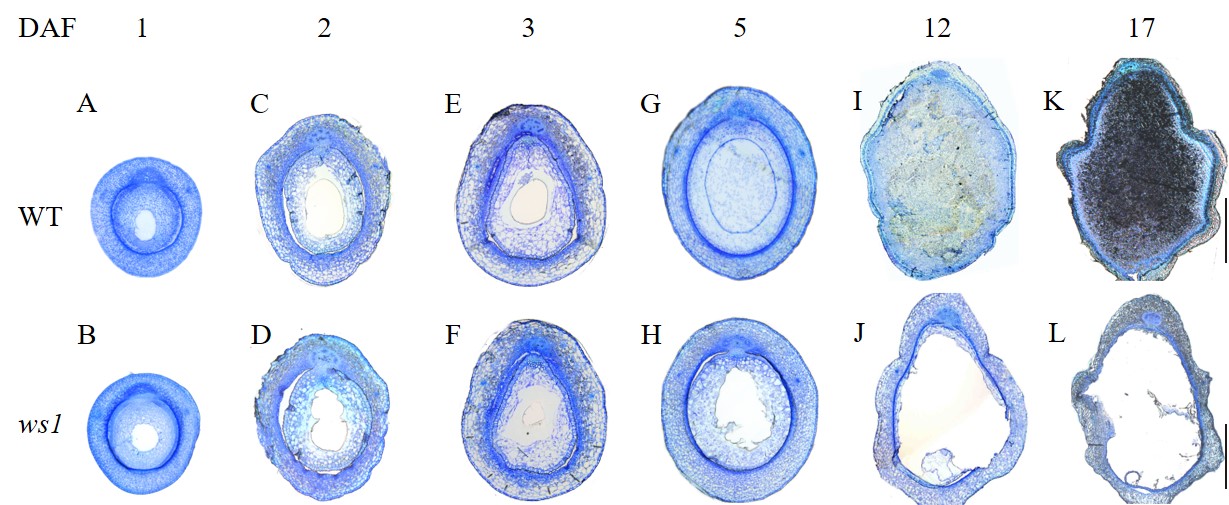
**

**Fig. S3 Compares the developing caryopses from WT and *ws1-1*.**

Transverse sections of WT and *ws1-1* caryopses at different developmental stages. No visible difference was observed between *ws1-1* and WT seeds at 1 DAP but became obvious from 3 DAP onwards. At later developmental stages, while WT caryopses were filled with endosperm, *ws1-1* caryopses expanded without forming endosperm but remaining hollow inside. DAF, days after pollination; WT, wild type. Bar = 1.5 mm.


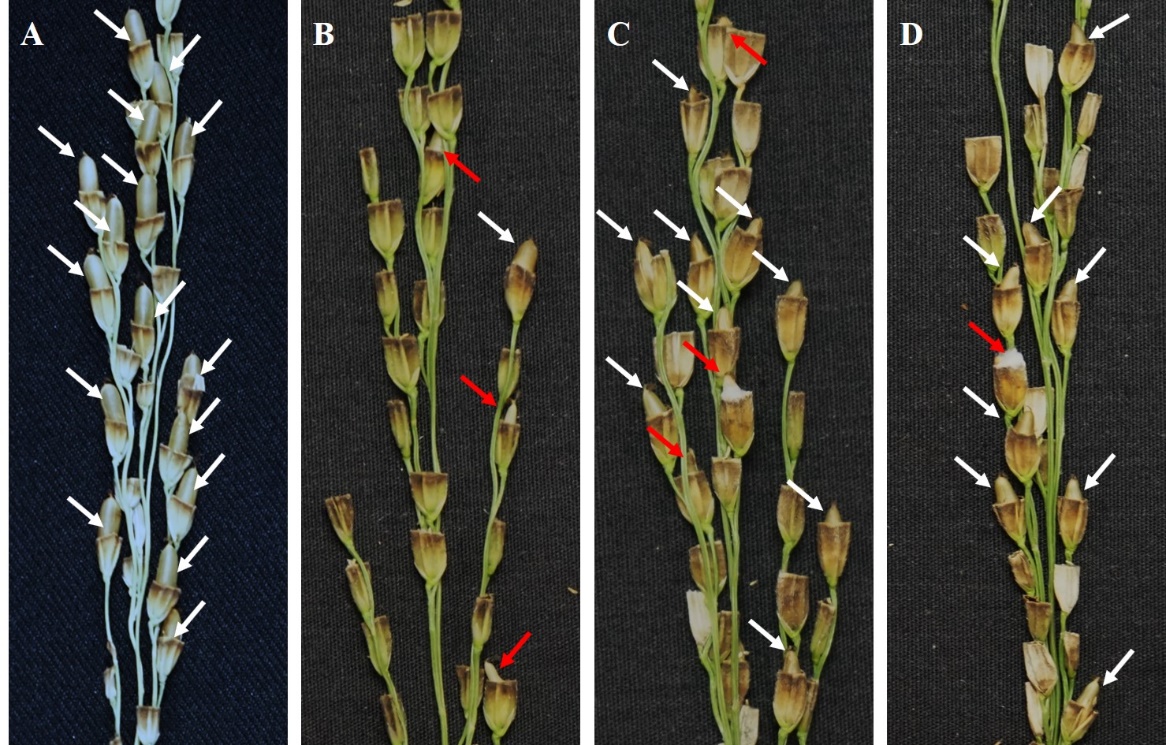


**Fig. S4** **Results of artificial fertilization within or between WT and *ws1-1*.**

(A) WT selfing. (B) *ws1-1* selfing. (C) *ws1-1* × WT. (D) WT × *ws1-1*. The white arrows and red arrows indicate starchy seeds and watery seeds, respectively.

**
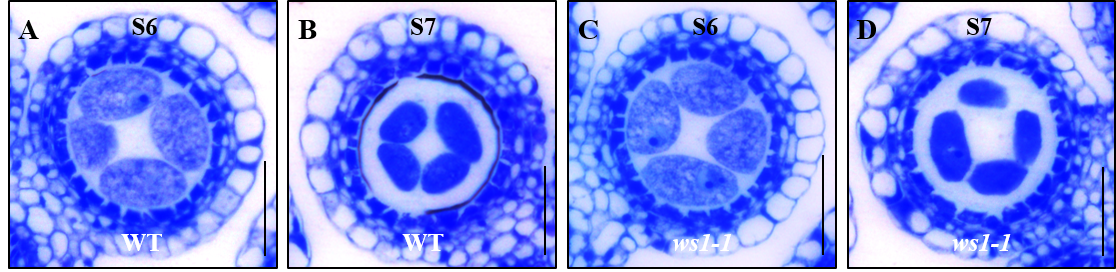
**

**Fig. S5 Comparison of microsporogenesis at stages 6 and 7 in WT and mutant anthers*.***

Sections stained with 0.1% toluidine blue. The development stages were described by Zhang et al. (2011). Scale bars = 25 µm.

**
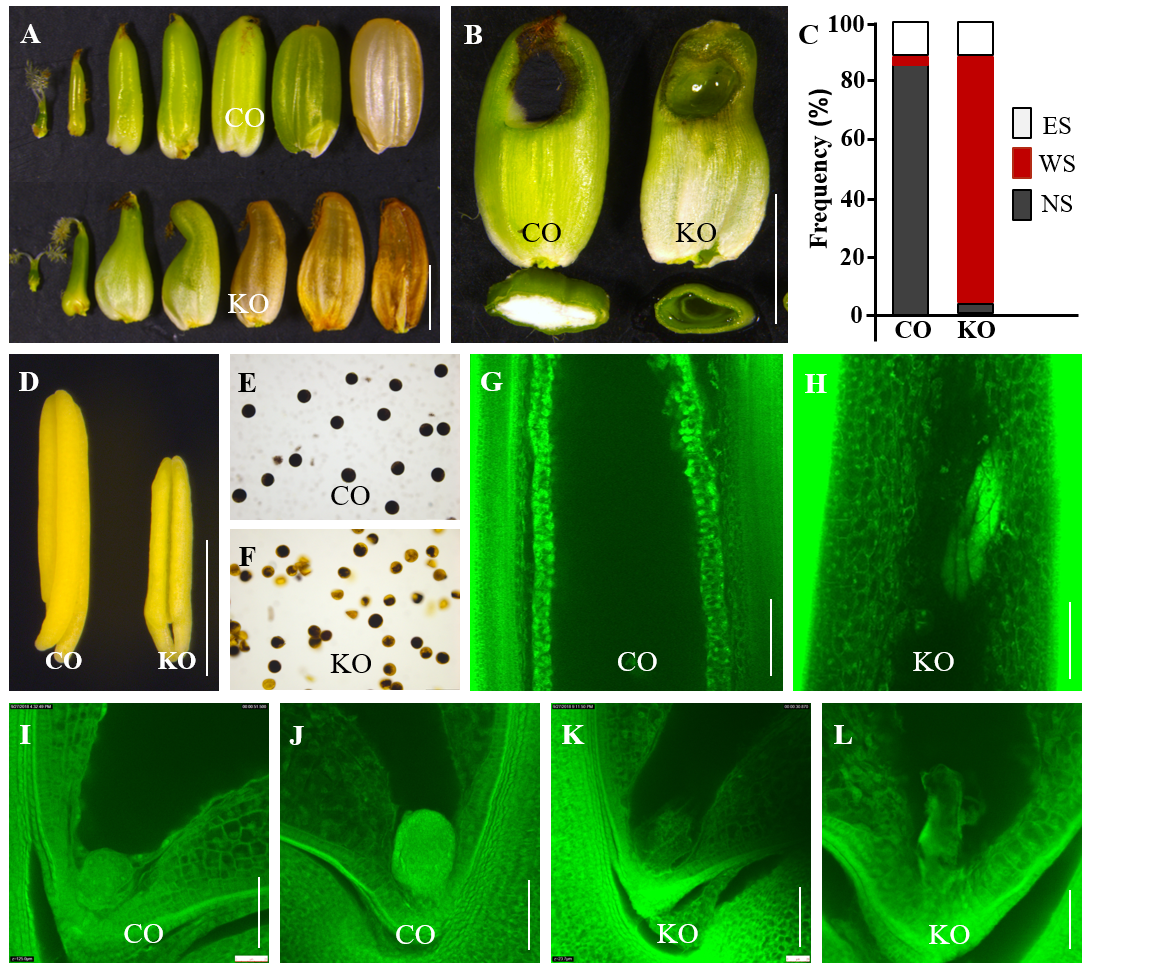
**

**Fig. S6 The phenotypes of the seeds, anthers and pollens, and the early seed development in** **the complementary and knockout plants.**

A Seed phenotype of the genetic complementatary and knockout plants at different DAFs. B The starchy endosperm in the complementary seed and the transparent liquid in knockout seeds (stained with I_2_-IK solution). C Cumulative percentages of normal seeds (NS), watery seeds (WS), and empty seeds (ES, containing no developed caryopsis) in the complementary and knockout plants at the mature stage were obtained from statistics of 2000 grains each. D-F Anthers and pollen grains in the complementary and knockout spikelet at the mature stage; the pollens were stained with I_2_-IK solution (normal pollens showed dark color). G, H The distribution of the endosperm cells in the complementary and knockout plants. I-L The early-stage embryo in the complementary and knockout plants. CO, genetic complementation plant; KO, knockout mutant. Scale bars = 2 mm (A, B, D) or 50 µm in (G-L).


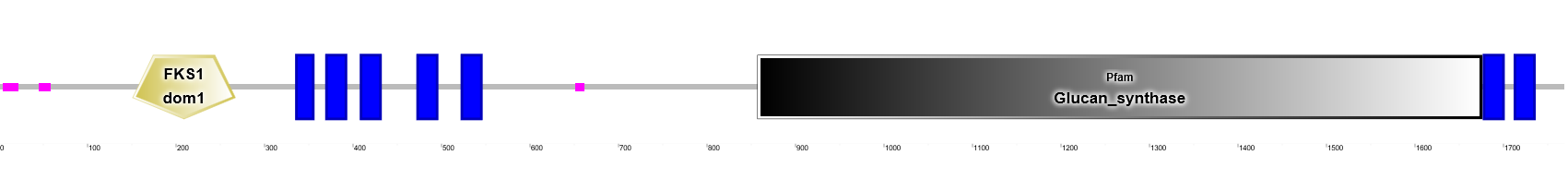


**Fig. S7 The predicted protein structure of WS1/OsGSL2.**

**
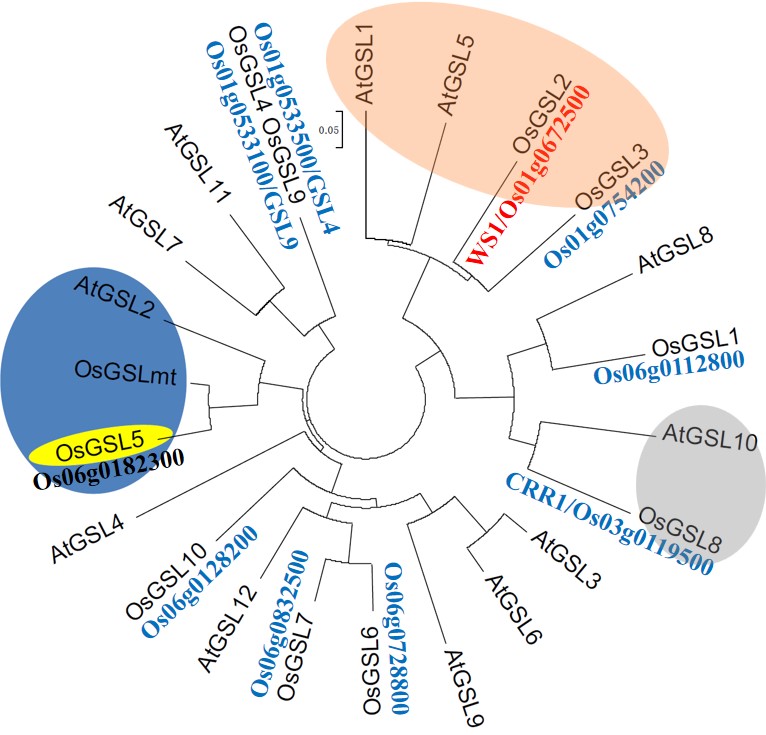
**

**Fig. S8 WS1 is the homolog of AtGSL1 and AtGSL5 (**Revised according to Shi et al. 2015).

**
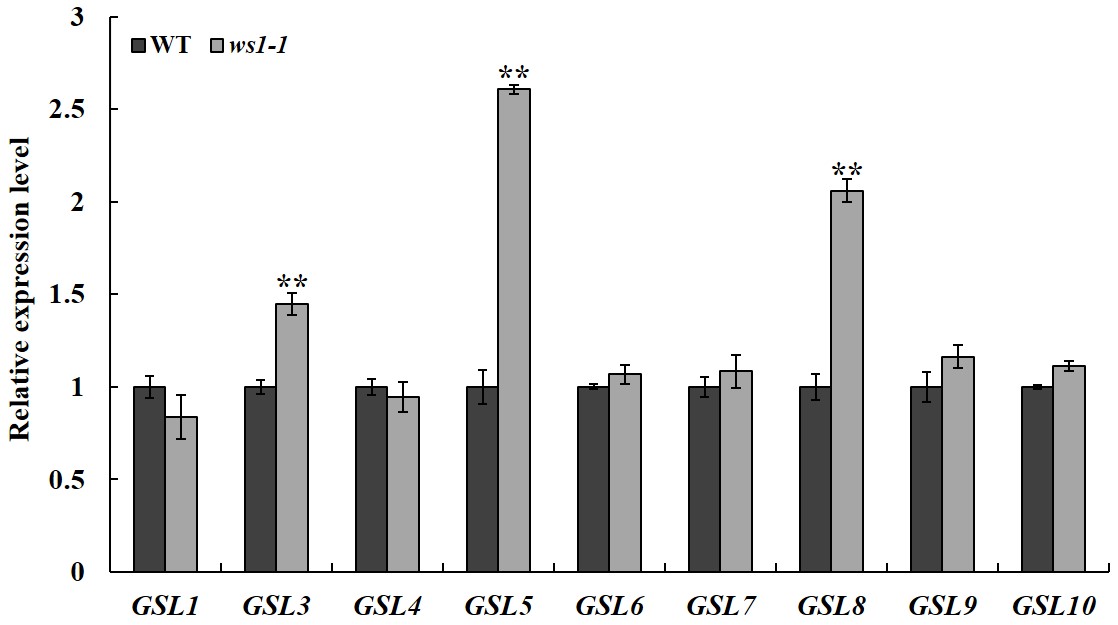
**

**Fig. 9 RT-qPCR analysis of *OsGSL* genes in WT and *ws1-1* anthers at microspore development.**

Error bars represent the SD of three biological replicates. Asterisks indicate significant differences between the WT and *ws1-1* according to Student’s t-test (*P<0.05 and **P<0.01).
