# Supplementary material for: Glucan Synthase-like 2 is Required for Seed Initiation and Filling as Well as Pollen Fertility in Rice
Source: Rice (N Y). 2023 Oct 7;16:44. doi: 10.1186/s12284-023-00662-z (PMC10560172; doi:10.1186/s12284-023-00662-z)
Supplement: Supplementary file 2 — Additional file 2: Table S1 Primers used in this study. Table S2 Results of WS1 sequence editing by Cas9-induced knockout. [file 12284_2023_662_MOESM2_ESM.docx]

**Additional file 2**

**Table S1** Results of *WS1* sequence editing by Cas9-induced knockout.

Del, deletion; Ins, insertion; ORF, Open reading frame; PTT, premature transcription termination.

| Alleles | lines | DNA sequence | ORF | Protein |
| --- | --- | --- | --- | --- |
| WT | CK | gcgccgatctgctcttcgcggggctccac | 5310 base | 1769 aa |
| *ko-1* | 1-3 | gcgccgatctgctcttcgacggggctccac +1 bp | Ins 470^th^, PTT | 156 + new 90 aa |
| *ko-2* | 4-7 | gcgccgatc~~tgctc~~ttcgcggggctccac -5 bp | Del 461^st^-466^th^, PTT | 153 + new 91 aa |
| *ko-3* | 8-13 | gcgccgatctgct~~cttc~~gcggggctccac -4 bp | Del 465^th^-468^th^, PTT | 155 + new 147 aa |
| *ko-4* | 14-16 | gcgccgatctgc~~t~~cttcgcggggctccac -1 bp | Del 464^th^, PTT | 154 + new 150 aa |

**Table S2** Primers used in this study.

| Name | Primer sequence (5′-3′) | | | Purpose | |
| --- | --- | --- | --- | --- | --- |
|  | Forward | Reverse | |  |  |
| RM543 | ctgctgcagactctactgcg | aaatattacccatccccccc | Mapping | |  |
| RM488 | cagctagggttttgaggctg | tagcaacaaccagcgtatgc |  |  |  |
| InDel 1 | aattcagttgagcgccgttt | tcccgaggaggcgtattttt |  |  |  |
| InDel 2 | gcagagtcacgagcctatct | tggtgcgtttgtgtcattga |  |  |  |
| InDel 5 | tcctatggcttgcgatgctt | tgtatggcgtttctgctgga |  |  |  |
| InDel 6 | cttacctttcagactcagtgcg | ttttgcaccctggtttcacat |  |  |  |
| InDel 8 | gagaaccgaagtctttgttagca | catgccgttccaccccat |  |  |  |
| GUS | ctagtggcaacggtgagcaagtggcaacggtg | gctccacaacaaaaccacggcacaacaaaacca | Vector construction | |  |
| Complementation | gagctcggtacccggggatccagtcccgactctctcccaaa | acgacggccagtgccaagctttgcaacgtttcgtgtgagtt |  |  |  |
| Knockout  GFP | agctcctccgcaactacac  acgggggacgagccggtaccatgacgacgccgcgggcc | gtagatgggcgtgacgacg  gcccttgctcaccatgtcgacaactccatgtgatttttttccagt |  |  |  |
| ACTIN | gattgccaaggctgagtacga | aaaagagagaaacaagcaggagga | RT-qPCR | |  |
| OsGSL1 | caactccgaagaagtcaacc | tacatccgagcaatctcacg |  |  |  |
| OsGSL2 | ctagtggcaacggtgagcaa | gctccacaacaaaaccacgg |  |  |  |
| OsGSL3 | gtggcacgtttgtatgagat | ctaactgcgataaccttgtt |  |  |  |
| OsGSL4 | ttggcattcatccctactgg | tacatcctcgccatctcctg |  |  |  |
| OsGSL5 | ggtcctgaaggtggtatcca | ggcttgctggcttgagatat |  |  |  |
| OsGSL6 | gccatccctcacatgacagt | gacacgaaaggaaaccaagc |  | |  |
| OsGSL7 | atgacagtccaggacatctt | gatacaaacgggaaccaagc |  | |  |
| OsGSL8 | gtcattgccaagttagttgctgtagcag | gccactcatatcaacagacagcacttc |  | |  |
| OsGSL9 | gtcattgccaagttagttgctgtagcag | gccactcatatcaacagacagcacttc |  | |  |
| OsGSL10 | tgtggcgtttgcgatcttg | gctccacaacaaaaccacgg |  | |  |
|  | | | | |  |
